# Noncanonical DNA Motifs as Transactivation Targets by Wild Type and Mutant p53

**DOI:** 10.1371/journal.pgen.1000104

**Published:** 2008-06-27

**Authors:** Jennifer J. Jordan, Daniel Menendez, Alberto Inga, Maher Nourredine, Douglas Bell, Michael A. Resnick

**Affiliations:** 1 Laboratory of Molecular Genetics, National Institute of Environmental Health Sciences, NIH, Research Triangle Park, North Carolina, United States of America; 2 Curriculum in Genetics and Molecular Biology, University of North Carolina at Chapel Hill, Chapel Hill, North Carolina, United States of America; 3 Unit of Molecular Mutagenesis and DNA Repair, National Institute for Cancer Research, IST, Genoa, Italy; National Institute of Diabetes and Digestive and Kidney Diseases, United States of America

## Abstract

Sequence-specific binding by the human p53 master regulator is critical to its tumor suppressor activity in response to environmental stresses. p53 binds as a tetramer to two decameric half-sites separated by 0–13 nucleotides (nt), originally defined by the consensus RRRCWWGYYY (n = 0–13) RRRCWWGYYY. To better understand the role of sequence, organization, and level of p53 on transactivation at target response elements (REs) by wild type (WT) and mutant p53, we deconstructed the functional p53 *canonical* consensus sequence using budding yeast and human cell systems. Contrary to early reports on binding *in vitro*, small increases in distance between decamer half-sites greatly reduces p53 transactivation, as demonstrated for the natural TIGER RE. This was confirmed with human cell extracts using a newly developed, semi–*in vitro* microsphere binding assay. These results contrast with the synergistic increase in transactivation from a pair of weak, full-site REs in the MDM2 promoter that are separated by an evolutionary conserved 17 bp spacer. Surprisingly, there can be substantial transactivation at noncanonical ½-(a single decamer) and ¾-sites, some of which were originally classified as biologically relevant canonical consensus sequences including PIDD and Apaf-1. p53 family members p63 and p73 yielded similar results. Efficient transactivation from noncanonical elements requires tetrameric p53, and the presence of the carboxy terminal, non-specific DNA binding domain enhanced transactivation from noncanonical sequences. Our findings demonstrate that RE sequence, organization, and level of p53 can strongly impact p53-mediated transactivation, thereby changing the view of what constitutes a functional p53 target. Importantly, inclusion of ½- and ¾-site REs greatly expands the p53 master regulatory network.

## Author Summary

Within human cells, the tumor suppressor p53 is the central node of regulation required to elicit multiple biological responses that include cell cycle arrest and death in response to stress or DNA damage, where mutations in p53 are a hallmark of cancer. As a master regulatory gene, p53 controls the action of target genes within its network by directly interacting with a widely accepted consensus DNA binding sequence, composed of two decamer ½-sites that can be separated by up to 13 bases. While mismatches from consensus sequence are frequent, the canonical consensus sequence places a limitation upon the organization and number of target genes within the p53 transcriptional network. Using yeast and human cell systems, our goal was to further understand how the DNA sequence, DNA organization, and level of p53 expression might influence the inclusion of genes within the p53 regulatory network. We found that increases in spacer beyond a few bases greatly reduce responsiveness to p53. Importantly, we established that p53 can function from noncanonical sequences comprising only a decamer ½-site or a ¾-site. These findings further define and expand the universe of potential downstream target genes which may be regulated by p53 and bring further diversity into the p53 regulatory network.

## Introduction

The tumor suppressor p53 (OMIM no. 191170) is a sequence-specific *master regulatory gene* that controls an extensive transcriptional network providing for genome integrity in response to cellular and environmental stresses or damage [1]–[3]. p53 differentially regulates the expression of target genes, as well as microRNAs associated with cell cycle control, apoptosis, DNA repair, angiogenesis, senescence and carbon metabolism [4],[5]. A variety of factors such as stress and cell-type dependent, post-translation modifications and transcriptional co-factors can influence p53-induced transcriptional changes [6]–[8]. Paramount to p53 transcriptional function is the direct interaction between p53 and its targeted DNA sequence. The nature of this interaction could *per se* determine transactivation capacity, as well as influence p53-mediated biological processes [9]. Such activities are often altered during human cancer development as highlighted by the frequent appearance of p53 missense mutations in its sequence-specific DNA binding domain [10],[11] which can abrogate or alter p53 transactivational activity that result in changes in biological responses, such as the balance between apoptosis and survival in response to DNA damage. [3], [10], [12]–[15].

Through *in vitro* studies, a consensus p53 DNA binding sequence has been derived comprising a motif of two decamers (half-sites) RRRCWWGYYY (n) RRRCWWGYYY, (where R = purine, W = A or T, Y = pyrimidine and n is 0–13 bases) where each decamer is composed of two adjacent p53 monomer binding sites (quarter-sites) in inverted orientation [16]–[18]. p53 binds cooperatively to the consensus RE as a dimer of dimers, where a tetramer is the accepted functional unit required for full transcriptional activity [19]–[26]. Most functional response elements (REs) identified in association with p53 target genes depart from this consensus, where base changes are tolerated at each position with the exception of the C and G at positions 4 and 7 in each half-site [27],[28].

Recent crystal structures show that the interactions between p53 and DNA are influenced by the base pairs present in the RE sequence [29] while binding assays in solution established a wide range of dissociation constants among natural p53 REs [23]. Functional studies in model systems demonstrate that the RE sequence and amount of p53 expression can dramatically influence the level to which p53 can transactivate from a specific RE [3],[30]. However, results of those various approaches do not fully overlap and it is still not well-understood how the loose consensus, in terms of base variation and spacer length between half-sites affects p53 binding to DNA, or how differential binding relates to transactivation specificity [31]–[33].

More recently, several studies have refined the accepted, or canonical p53 consensus binding sequence based on presumed unbiased chromosome- or genome-wide *in vivo* binding assays using chromatin immunoprecipitation (ChIP) [34]–[37]. However, the findings may be skewed towards stronger p53 interacting sequences or influenced by the agent used to induce p53 protein, as well as the human cell lines examined. While ChIP provided a powerful tool for identifying sequences that p53 could bind and the results pointed to a refined p53 consensus with more restrictive sequence features [34], these assays could not address the strength of p53 binding. Furthermore, the experimental approach failed to retrieve known p53 binding sites, indicating that the technique could miss interactions between p53 and weaker response elements which might include noncanonical sites that do not match the accepted consensus motif.

Bioinformatics studies to identify p53 REs are guided by identified sequences. Based on the consensus sequence, computational algorithms have exploited the base pair composition of known p53 REs to determine binding probabilities of p53 towards sequences and generate position-weight matrices (PWM) or “logos” [38],[39]. While such algorithms may guide the identification of p53 binding sites in the genome, they cannot assess the strength of p53 binding to these sequences. The ability of p53 to bind (and presumably transactivate from) a sequence is often related to compliance with the canonical consensus sequence where each position in the motif is assumed to be equivalent and mutually exclusive in terms of affecting p53 binding [40]. However, PWM values appear not to be a good predictor of p53 transactivation [30].

While the canonical consensus sequence has guided studies of the p53 network, an additional layer of complexity was identified recently suggesting that the canonical motif is not limited to two decamers in human cells. p53 dependent-transactivation was detected in association with a decamer half-site created by a single nucleotide polymorphism (SNP) in the promoter of the *FLT-1* gene (the vascular endothelial growth factor (VEGF) receptor-1 gene) [41]. These results showing that p53 can function from a noncanonical consensus sequence imply that the number of potential p53 target sites within the genome may be much greater than anticipated. Current methods that identify and/or define putative p53 REs would overlook such noncanonical binding elements.

Stimulated by the observation of a transcriptionally active p53 half-site, as well as by the finding that even a single nucleotide change can greatly impact transactivation potential of a RE [42],[43], we have systematically deconstructed the canonical p53 RE sequence to address the requirements for a functional p53 binding site. Utilizing *in vivo* systems we developed in the budding yeast *Saccharomyces cerevisiae*, the transactivation potential of p53 was first examined from p53 canonical consensus REs containing base changes and/or variations in organization of binding motifs (*i.e.*, multiple REs and various spacer lengths) and then assessed transactivation from ½- and ¾-site REs, which we refer to as noncanonical REs. For these noncanonical REs the surrounding sequence does not resemble a p53 binding sequence and the motif itself would not be recognized within standard descriptions of a p53 canonical consensus sequence. Within the yeast system, we have addressed not only the ability of p53 to function from specific target sequences (*i.e.*, on/off), but also the extent of transactivation from these sites at variable levels of expression. These studies have been extended to transactivation capacity and p53 promoter occupancy in a human cell system, and the *in vivo* functionality evaluations were compared with results obtained in a recently developed semi-*in vitro* binding assay using human cell nuclear extracts. Several structural mutants were examined to assess the p53 structural requirements for transactivation from noncanonical REs. Overall, the findings expand our understanding, as well as the anticipated size of the human p53 master regulatory network.

## Results

### Isogenomic System To Address p53 Transactivation from REs in Diploid Yeast

Previously, we had developed a plasmid-based haploid yeast system to systematically evaluate the contribution of RE sequence and p53 expression level towards p53 differential transactivation [14],[30]. While many factors may ultimately determine p53 transactivation of individual genes in human cells including stress stimuli, post-translational modifications, and transcriptional cofactors, the yeast system addresses the potential for wild type and mutant p53 to bind and transactivate from various REs derived from human genes when placed in a constant chromatin environment.

**Figure 1:**
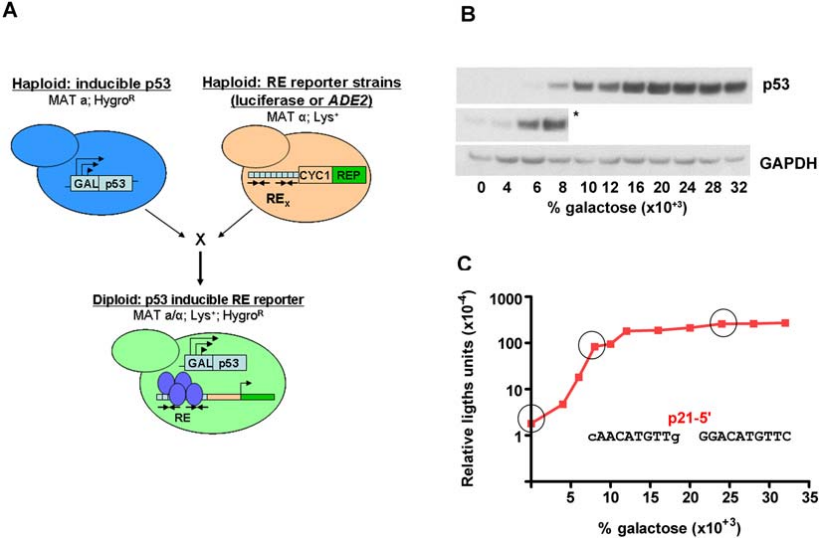
Isogenomic diploid yeast system to investigate transcriptional capacity of p53 towards many REs at various p53 levels. (A) Transactivation capacities of p53 (WT or mutant) from cognate REs were determined using diploid strains derived from haploids where p53 and reporters with upstream REs were integrated into different chromosomal loci. Within the *p53-host* strains, p53 (WT or mutant) was controlled by a “rheostatable” *GAL1* promoter (blue cells) (previously described [109]) which allows for controlled, inducible expression of p53. Reporter strains contained promoter REs upstream of the minimal *CYC1* promoter and either the *ADE2* color reporter (REP) or the firefly luciferase reporter (tan cells) [28]. Mating of the REP and *p53-host* strains results in isogenomic diploid yeast that provide for rapid assessment of the transactivation potential for WT or mutant p53 proteins towards many individual REs in the p53 transcriptional network [14]. (B) Inducible expression of p53 under the rheostatable *GAL1* promoter. The *GAL1* promoter allows for controlled expression of p53 depending on the level of galactose in the media. Raffinose was added to provide a basal level of expression, as the *GAL1* promoter is derepressed, but not induced in media containing this carbon source. Presented is a Western blot analysis of p53 expression 24 hours post-inoculation with raffinose or raffinose plus increasing amounts of galactose (0.004–0.032%). Increases in galactose from 0–0.032% correlated with an increase in p53 over a 100-fold range. The p53 protein was detected with DO-1 and pAb1801 antibodies. The asterisk (*) depicts a longer exposure to reveal protein at basal and lower galactose levels. GAPDH, identified by immunodetection provided a standard loading control. (C) Quantitative assessment of p53-induced transactivation from the p21-5′ RE *in vivo*. The diploid yeast strain containing *GAL1*::WT p53 crossed with the p21-5′ RE-luciferase REP was grown overnight in complete medium, diluted, washed and inoculated into selective medium containing either raffinose or raffinose plus increasing galactose (0–0.032%) for 24 hours. Protein lysates were obtained and a luciferase assay was used to determine the transactivation capacity of p53 from the p21-5′ RE. The strength of transactivation was calculated as relative light units/µg protein. Circled are the basal, linear-increase, and plateau phases of the transactivation response as a function of galactose concentration and are referred to as basal, moderate and high levels of p53 expression.

We have expanded this experimental system to diploid yeast to further assess the transactivation capacities of p53 (WT or mutant) (Figure 1A). Two panels of modified *S. cerevisiae* strains were generated. The first was a set of p53 host strains in which p53 (WT or mutant) is directed by a “rheostatable” *GAL1* promoter that allows for controlled, over 200-fold, inducible expression of p53 in yeast depending on the carbon source in the media (Figure 1B and Figure S1). Importantly, similar to p53 protein accumulation in mammalian cells following stress [44], expression from the *GAL1* promoter in yeast displays a graded transcriptional response such that there is a range of activity from the promoter as opposed to a binary, or on/off response [45]–[47]. Biggar and Crabtree [45] demonstrated through fluorescence-activated cell sorting (FACS) experiments that expression of green fluorescent protein from a *GAL1*-GFP reporter within a population of cells generated a single fluorescent peak where the intensity of the peak was dependent upon the concentration of galactose supplemented in the media. Thus, increases in galactose will result in a homogeneous response where the vast majority of cells in the population respond (within our system expressing an induced amount of p53) rather than merely increasing the percentage of cells within the population expressing the maximal level of protein.

The second set of strains of opposite mating type contained promoter REs upstream of the minimal *CYC1* promoter and either the *ADE2* color reporter or the firefly luciferase reporter [28]. To facilitate the construction of a large number of p53 mutants and REs at chromosomally located target loci we employed the *delitto perfetto* system for *in vivo* mutagenesis. *Delitto perfetto* utilizes oligonucleotides and targeted homologous recombination to rapidly generate *S. cerevisiae* yeast strains with specific genetic alterations [48],[49].

Mating of the reporter and p53 host strains results in isogenic, diploid yeast that enable the rapid assessment of the transactivation potential for WT or mutant p53 proteins towards many individual REs in the p53 transcriptional network [14]. Importantly, all the conditions in the cells are constant, i.e., isogenomic, where the only variables between strains are the RE sequence, WT or mutant p53 and level of expression.

The luciferase reporter provided a quantitative estimate of transactivation capacity of WT p53 from the various REs in cultures of logarithmically growing cells. The strength of transactivation (relative light units/µg protein) from each RE were compared to transactivation from the strongly transactivated p21-5′ RE at high p53 expression (0.024% galactose). An example of transactivation capacity of p53 over a range of expression is shown by the quantitative assessment of p53-induced transactivation from the p21-5′ RE *in vivo* (Figure 1C). Functional assessment with the luciferase reporter assay provided an indication of the kinetics of transactivation with a basal level of transactivation at 0.00% galactose (2% raffinose), initial induction of transactivation (between 0.004–0.008% galactose) and maximal level of transactivation (between 0.016–0.024% galactose).

### Increases in Distance between Weak, Full-Site REs Can Lead to Synergistic Transactivation

A common feature of many p53 target genes, including p21, PUMA, BAX, and DDB2 (p48), is the presence of multiple p53 binding sites [50]–[56]. The distance between binding sites is variable and can even be overlapping. The *Mdm2* gene, which targets p53 for degradation by the ubiquitination pathway through a negative feedback loop, is an example of a p53 target gene that contains two promoters. The upstream promoter is constitutively active and does not rely on p53 for transactivation, whereas the second is in the first intron and is p53-dependent [57],[58]. The second promoter contains two full-site p53 REs separated by 17 nucleotides (nt). Using our diploid yeast-based p53 rheostatable system, we investigated the interaction between these two REs and the impact of this spacer by changing the distance between the murine MDM2 REs.

**Figure 2:**
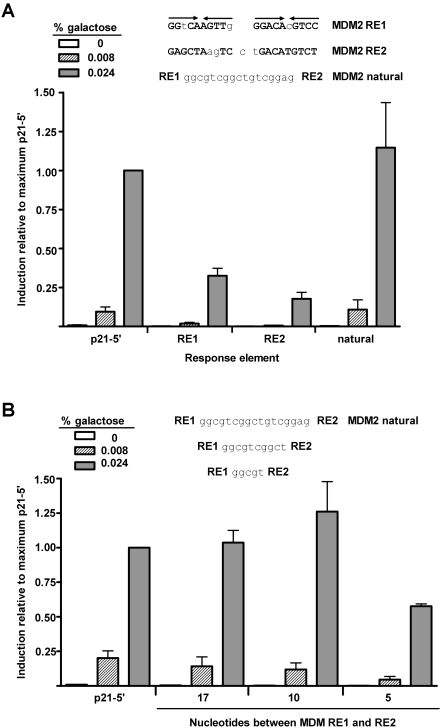
Weak REs can function synergistically when separated by a spacer. (A) To ascertain if p53 functions from the two full-site REs of MDM2 independently or if the REs interact, p53 transactivation from the isolated REs, as well as the natural MDM2 RE containing a 17 nucleotide spacer were evaluated. Isogenomic diploid yeast strains containing the p21-5′ and MDM2 REs, as indicated, were grown in increasing concentrations of galactose to induce p53 protein to basal, moderate and high levels of expression. The ability of WT p53 to transactivate from RE sequences was measured by a luciferase assay 24 hours after inoculation into the galactose supplemented media. Induction from each RE was compared relative to the ability of p53 to transactivate from the p21-5′ RE at 0.024% galactose and is depicted as the mean and standard error of measurement (SEM) of 6 independent experiments. The average light units/µg protein from p21-5′ at 0.024% galactose was 2.1 million. Solid arrows over the sequences indicate a ¼-binding site. (B) Impact of reducing the spacer to 10 and 5 nucleotides. The average light units/µg protein for p21-5′ at 0.024% galactose was 2,800,000.

The induction from the individual MDM2 REs at high p53 expression was much weaker than observed with the strong p21-5′ RE (Figure 2A): ∼33% (RE1) and ∼18% (RE2) relative to p21-5′ RE. However, transactivation from the natural MDM2, containing the 17 base spacer, was much higher than the sum of the individual REs, reaching the p21-5′ RE levels. The synergy was apparent at both moderate and high p53 levels. To determine the impact that a spacer may play for full-site REs, the distance was reduced to either 10 or 5 nt. As shown in Figure 2B, decreasing the separation to 10 nt had little effect. However, a decrease to 5 nt resulted in a substantial reduction in transactivation suggesting that synergistic interactions are lost as full REs become closely spaced, although transactivation from the two sites remain additive.

### Increase in Spacer between Half-Sites Decreases p53 Transactivation

**Figure 3:**
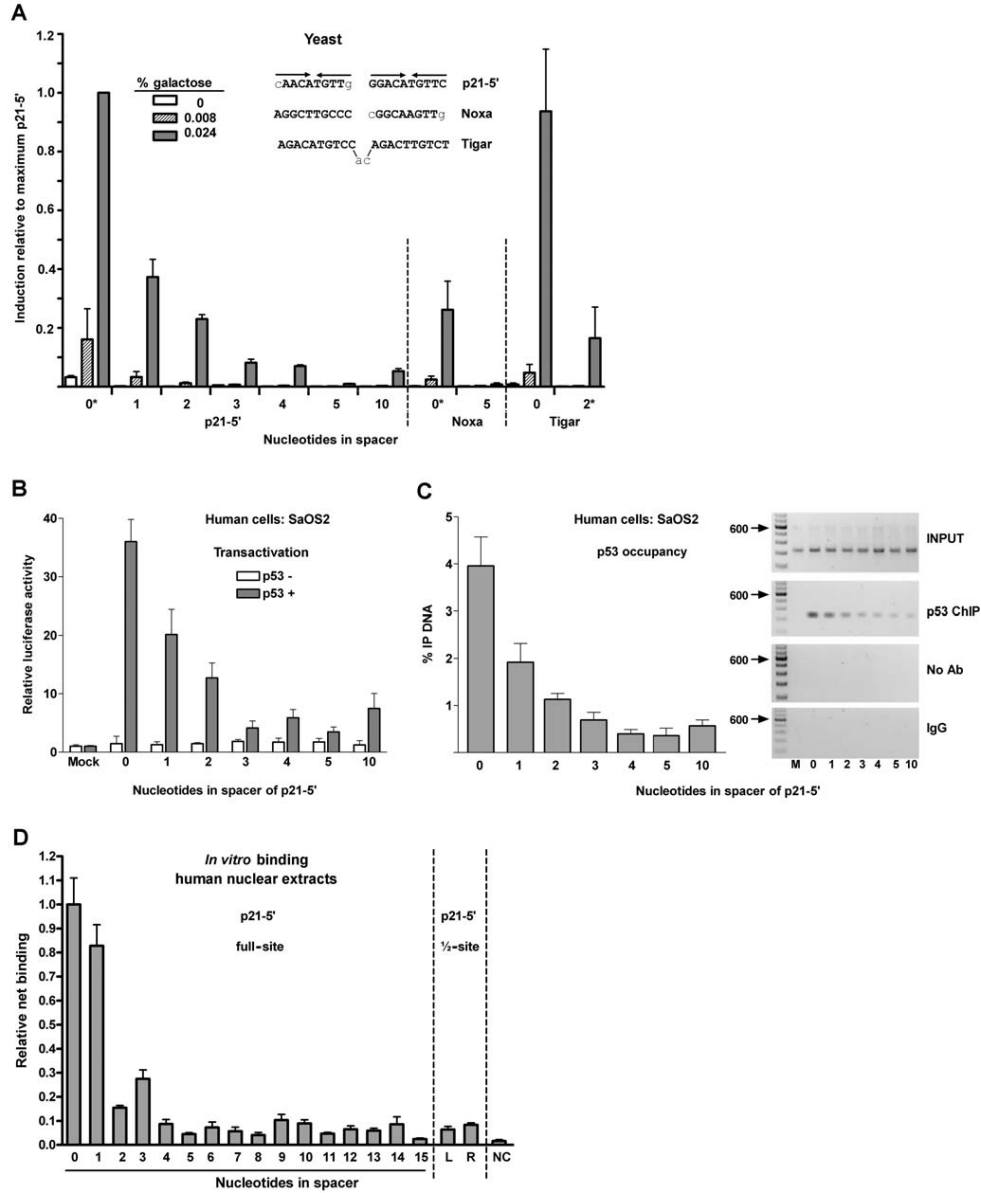
Spacer decreases p53 transactivation, promoter occupancy and binding in yeast and mammalian cells and *in vitro*. (A) Transactivation in yeast. The ability of p53 to transactivate from REs containing spacers of variable nucleotide length sequences was measured 24 hours after p53 induction with a quantitative luciferase assay. Induction from each RE at various p53 expression levels were compared to the induction from the p21-5′ RE at 0.024% galactose. The average light units/µg protein for WT p53 towards p21-5′ at 0.024% galactose for a minimum of 6 biological repeats was 1.26 million. *indicates the number of nucleotides in the spacer of the natural RE. Solid arrows identify a ¼-binding site. (B) Human SaOS2 cells were transfected with p21-5′::luciferase reporter constructs containing spacers of increasing length between decamer half-sites in the presence (solid bars) or absence (open bars) of the high expressing pCMV-p53 WT vector. At 48 hours post transfection, induction of the luciferase reporter was assessed. Relative luciferase activity was compared to the pGL3-P plasmid lacking the p53 RE (mock) and is represented by the average and standard deviations of three independent experiments, each containing three replicates. (C) Occupancy of p53 at p21-5′ REs in human cells. The p21-5′ promoter constructs containing the increasing spacers between half-sites were co-transfected with p53 into SaOS2 cells (as described in (B)). Twenty-four hours later, ChIP analysis was performed. Presented are the average and standard deviation from 4 independent experiments (left). PCR products of the Input DNA (input) and ChIP DNA (p53, IgG, or no antibody, Ab) are shown (right). The “M” corresponds to a pGL3-P plasmid control lacking the p53 RE. No bands were observed above the 600 bp markers. (D) *In vitro* fluorescent microsphere binding assay to evaluate sequence-specific p53-DNA binding interactions (see Materials and Methods). Fluorescent microspheres bearing double stranded DNA fragments were multiplexed and incubated in the presence of nuclear extracts from non-treated (NT) or Doxo-treated (0.6 ug/mL [1 mM] Doxorubicin for 18 hours at 37°C) lymphoblastoid cells. The DNA fragments contained the p21-5′ RE, p21-5′ RE with spacers of increasing length (0–15), p21-5′ half-site RE (left or right), or a scramble sequence.

While weak full-site REs can interact synergistically when separated by 17 bases, previous studies have indicated that spacers between decamer half-sites of the canonical consensus RE can alter the ability of p53 to transactivate from a RE [18],[59],[60]. We systematically investigated the role that spacers may play on p53 transactivation using the yeast-based rheostatable promoter and a system based on expression following transfection into human cells. Addition of a one-base spacer between the p21-5′ half-sites resulted in a dramatic 60% decrease in p53 transactivation (Figure 3A) in yeast. Addition of a second nucleotide further decreased p53 transactivation to approximately 25% of transactivation at high p53 expression. Importantly, at lower p53 levels transactivation was essentially abolished, demonstrating that the impact of spacer is markedly affected by p53 expression level. Further increases in spacer resulted in decreased transactivation and at 5 nt there was almost no detectable luciferase activity at high p53 expression levels. Similar findings were observed in a plasmid-based haploid yeast system when p53 transactivation was measured from p21-5′ REs containing a spacer of 0, 2, 5, or 10 nt (Figure S2A) [3],[28],[30]. Thus, the length of spacer sequence between decamer half-sites combined with level of p53, greatly influences the ability of p53 to transactivate from the p21-5′ sequence. Interestingly, transactivation by the p53 family members p63β and p73β was also compromised when p21-5′ RE contained spacers (Figure S2B); a 2 nt spacer essentially abolished transactivation.

To determine the effect of spacers upon weak target REs in the p53 transcriptional network, transactivation was assessed from the RE of the human apopotosis gene Noxa [61] with and without a 5 nt spacer between the decamer half-sites. In comparison to the p21-5′ RE, transactivation from the Noxa RE was ∼30% of the levels of p21-5′ at high p53 expression (Figure 3A). This was comparable to p53 transactivation from the p21-5′ RE containing a spacer of 1 or 2 bases. A spacer of 5 bases between the Noxa half-sites abolished p53 responsiveness (also found with the haploid yeast system; Figure S2A).

Finally, we wanted to establish the impact that naturally occurring spacers in REs might have on transactivation. TIGAR (TP53 induced glycolysis and apoptosis regulator) contains a p53 target RE with a two-base spacer between decamers. This gene reduces levels of glycolysis, decreases free reactive oxygen species and attenuates the apoptotic response [62]–[64]. Interestingly, the natural TIGAR RE is one of the few examples in the genome where the binding element matches the canonical p53 consensus sequence precisely (i.e., no mismatches). However, p53 could only induce transactivation from this sequence to ∼20% of the levels with the p21-5′ RE, which does not precisely match the consensus p53 sequence (Figure 3A). When the spacer was removed, p53 could transactivate from TIGAR to levels comparable to the p21-5′ RE. Similar results were obtained with the haploid yeast system (Figure S2A).

The impact of spacers on the ability of p53 to function from a RE was also assessed in human cells under conditions of high p53 expression. Luciferase reporter vectors containing the p21-5′ RE with spacers of increasing length were generated in the vector pGL3 promoter (pGL3-P). The ability to transactivate from transfected p21-5′ REs was assessed in p53 null SaOS2 cells (derived from a human osteosarcoma line) that were transfected with a cytomegalovirus (CMV) based p53 expression plasmid [65]. Consistent with the results observed in yeast, p53-dependent transactivation decreased with increasing spacer length between decamer half-sites. As shown in Figure 3B, expression of WT p53 resulted in an ∼35-fold induction of transcription from the natural p21-5′ RE as compared to transfection with a p53 deficient plasmid, whereas transactivation from the p21-5′ RE containing a one- or two-base spacer resulted in a 45% and 67% reduction in relative luciferase activity, respectively. Similar to the situation in yeast, additional nucleotides resulted in >90% net reduction in transactivation. It is interesting that within the three systems–haploid and diploid yeast and human cells–transactivation from a RE with a spacer of 10 bases was slightly increased in comparison to a RE with a 5 base spacer.

### Spacers between Half-Sites Impact p53 Binding *in vivo* and *in vitro*

To determine if the difference in p53-dependent transactivation from REs containing spacers is simply due to a reduction in p53 promoter binding, we investigated *in vivo* occupancy using chromatin immunoprecipitation (ChIP) assays and *in vitro* binding using a newly developed microsphere binding assay (Noureddine et al., submitted). ChIP assays were performed on the luciferase reporter plasmids containing the p21-5′ RE with spacers of varying lengths which had been transfected into SaOS2 cells along with the p53-expressing plasmid. As shown in Figure 3C, a one nt spacer decreased occupancy at the p21-5′ RE by two-fold. Further increases in spacer length reduced p53 occupancy. No occupancy was observed in mock-transfected cells. Thus, the pattern of occupancy by p53 mirrored that for transactivation. Consistent with the transactivation results, p53 occupancy at the p21-5′ RE with a spacer of 10 nucleotides was slightly increased in comparison with a spacer of 5 nucleotides (0.6% vs. 0.4%).

To further characterize the impact of spacer on p53 interactions with a RE, we utilized a fluorescent microsphere binding assay to evaluate sequence-specific p53-DNA binding interactions (Noureddine et al., submitted). Briefly, this assay addresses p53 binding to individual beads with specific RE test sequences, where each bead “type” (*i.e.*, beads with specific REs) is identified with a unique double stranded oligonucleotide 24-nt “tag” sequence. Several bead types are then multiplexed in a binding assay and analyzed using Luminex technology to determine the amount of p53 bound to each bead type. We generated a series of oligos with the p21-5′ RE sequence of interest that contained various spacer lengths flanked by non-specific DNA. Each of these RE sequences was conjugated to beads. The bead types were combined and incubated for 60 minutes with nuclear cell extracts obtained from human lymphoblastoid cell lines that were either not induced or induced for p53 expression with doxorubicin (Dox). The level of p53 expression was approximately 15-fold greater in extracts from Dox-treated versus nontreated cells (data not shown). The p53 interaction with each bead type (*i.e.*, each p21-5′ RE variant) was determined after incubation with p53 antibodies and secondary antibodies conjugated with phycoerythrin.

As displayed in Figure 3D, the mean relative binding of p53 to the p21-5′ RE with a spacer of 1 nucleotide (0.83±0.09) was comparable to that for the p21-5′ RE (1.0±0.11) with no spacer. However, p53 binding to the p21-5′ REs was affected when the spacer between half-sites was ≥2 nucleotides, with a dramatic decrease at >4 nucleotides. The sequence of the spacer used to increase the distance between the decamer half-sites had no apparent effect on binding activity (data not shown). Furthermore, p53 did not bind to the negative control (NC) sequence which had the p21-5′ RE replaced with a scrambled sequence. Interestingly, p53 displayed the same residual binding to the individual half-sites of the p21-5′ RE [p21-5′ left (L) and right (R)] as it did towards REs containing a spacer of 4 nucleotides or more (0.06 and 0.08, respectively).

### p53 Can Transactivate from Half-Site REs in Yeast and Human Cells

**Figure 4:**
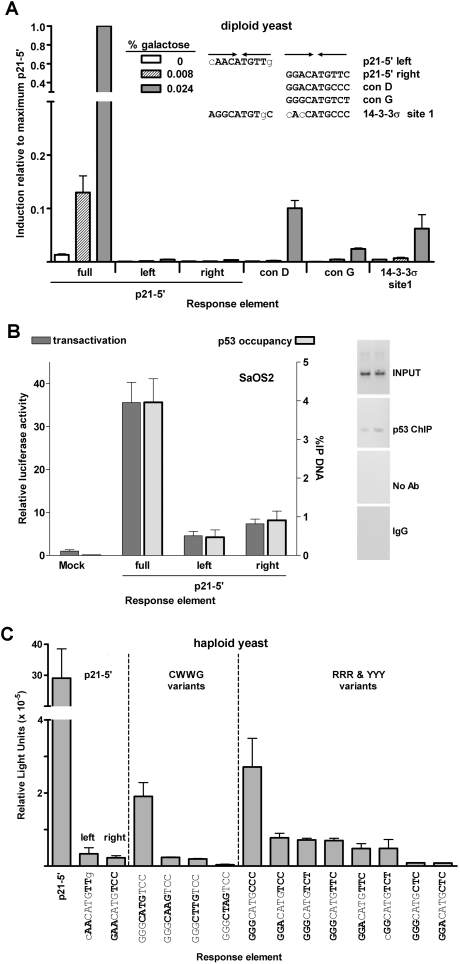
Half-sites function as noncanonical REs for transactivation in a sequence-dependent manner in yeast and human cells. (A) Transactivation from decamer REs in the diploid yeast was quantified with a luciferase assay. Protein lysates were obtained 24 hours post inoculation into galactose supplemented media. The average light units/µg protein for WT p53 towards p21-5′ at 0.024% galactose for six biological repeats was 1.8 million. Solid arrows indicate ¼-binding sites. Comparisons were made with transactivation from the p21-5′ full site at high protein levels. (B) Transactivation in human SaOS2 cells. The cells were co-transfected with WT p53 along with either the full, left or right half-sites of the p21-5′ RE containing reporter construct. Transactivation was assessed with the luciferase assay 48 hours later. Relative luciferase activity was compared to a mock transfection containing the promoter-less pGL3 plasmid. Presented are the averages and standard deviations of 3 independent experiments that were each done in triplicate. PCR products of the input DNA (input) and ChIP DNA (p53, IgG or no antibody, Ab) are shown (right). Input and ChIP PCR products for the mock and p21-5′ full-site are shown in Figure 3C. No bands were observed above the 600 bp markers. (C) Sequence dependence of p53 transactivation from decamer half-sites. The extent to which p53 transactivation from half-sites is sequence dependent at high expression levels (2% galactose) was determined with a plasmid-based haploid yeast system [30]. Relative light units/µg protein from ½-sites were compared to transactivation from the yLFM strain containing the p21-5′ full-site RE. The average light units/µg protein from p21-5′ at 2% galactose was 2.9 million.

Since a low level of binding or transactivation was observed with widely separated decamers, we investigated p53 binding and transactivation from single decamer sequences in the yeast diploid and human cell systems. Transactivation was barely detectable from the left or right decamers of the p21-5′ RE (Figure 4A). However, two additional complete consensus half-sites, designated Con G and Con D, were able to support transactivation at a level corresponding to 2.4% and ∼10%, respectively, of that from the full p21-5′ RE. Importantly, transactivation was only observed at high levels of p53 expression. By way of comparison, transactivation from the con D half-site was comparable to levels obtained from the low-responding 14-3-3σ RE.

To assess the ability of p53 to transactivate from decamer half-sites in a mammalian system, luciferase reporter vectors containing the p21-5′ RE half-sites were transiently transfected with or without a vector containing p53 under the CMV promoter into p53 null SaOS2 cells [65]. As shown in Figure 4B (also see Figure 3B), WT p53 induced transactivation from the full p21-5′ RE was 35-fold greater than with an empty pGL3-P vector. However, there was clear induction from the left and right half-sites, 4- and 6-fold, respectively. These results correlated well with the relative p53 occupancy assessed by ChIP: 4% for the full RE, 0.4% for the left decamer and 0.9% for the right decamer and agree with the previous findings in Menendez et al. [66] that p53 can function from a half-site RE. Based on these findings, the p53 transcriptional network may incorporate more downstream targets than previously predicted.

### p53 Transactivation from Decamer Half-Sites Is Sequence-Dependent

The sequence requirements for p53-dependent transactivation were examined further, utilizing a plasmid-based haploid yeast system with rheostatable p53 expression (previously used to assess transactivation capacity of canonical full-site REs; see Materials and Methods and [30]). Similar to results from mammalian cells, p53 was able to weakly transactivate from p21-5′ half-site REs at a level that was ∼1% of the levels of transactivation from the p21-5′ full-site RE (Figure 4C). This result differed somewhat from that of the diploid yeast system where transactivation from the p21-5′ half-site REs was <0.5% of the transactivation from the full-length p21-5′ RE (Figure 4A). Transactivation from Con D was greater than p53 transactivation from either of the p21-5′ half-sites and Con D was greater than Con G in both systems.

We also examined the impact of changes in the WW of the core CWWG (W = A or T). The 3 bases on either side consisted of GGG and TCC which had been shown to enhance binding at full-site REs [33] (Figure 4C). Decamers required the central CATG motif at the junction of the monomer binding sites for modest levels of transactivation, ∼7% of the levels from the p21-5′ RE (Figure 4C). Altering the motif from CATG to CAAG or CTTG decreased transactivation another 2- to 3-fold whereas changing this motif to CTAG nearly eliminated transactivation.

Transactivation was also assessed with various combinations of sequences surrounding the CATG core domain. As shown in Figure 4C, transactivation with the GGG/CCC flanking sequence was comparable to the GGG/TCC sequence tested with the CWWG motifs. However, transactivation was reduced when other alterations were made. For example, a change in flanking sequence to GGG/CTC or GGA/CTC resulted in almost no transactivation.

### p53 Functionality from Noncanonical, ¾-Site REs in Yeast

**Figure 5:**
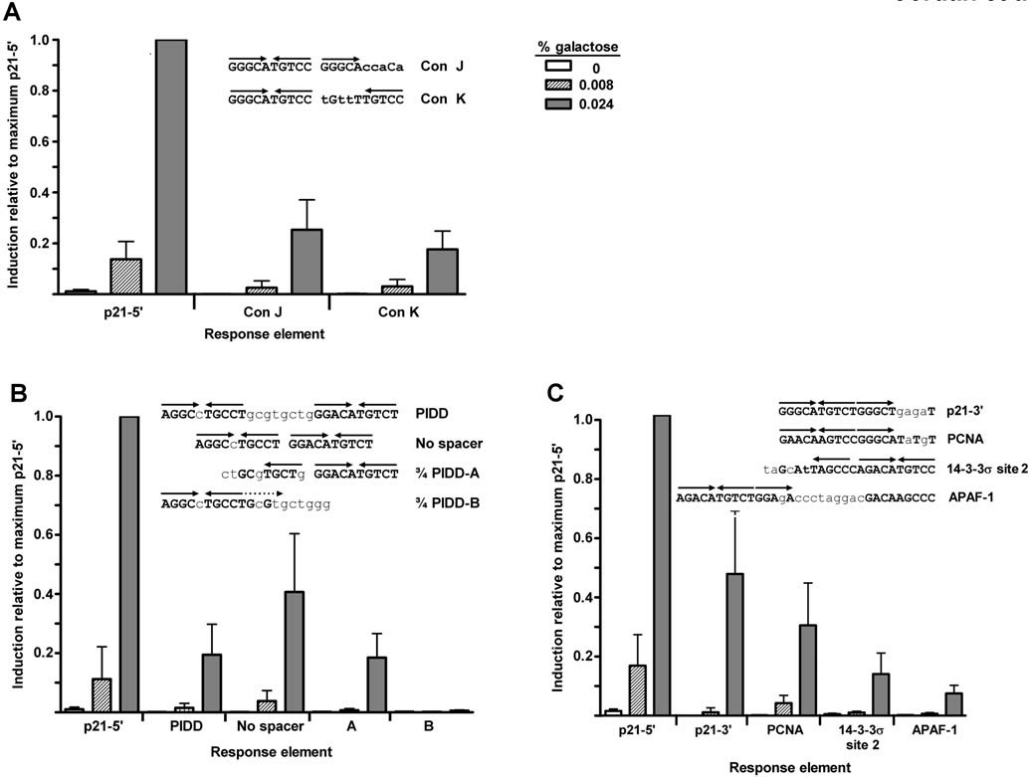
A ¾-site can function as a noncanonical RE in p53 transactivation. (A) Transactivation was assessed from ¾ REs Con J and Con K. The ability of WT p53 to transactivate was measured with the diploid yeast luciferase assay 24 hours post inoculation into the galactose-supplemented media. Induction from each RE was compared relative to the ability of p53 to transactivate from the p21-5′ RE at 0.024% galactose and is depicted as the mean and SEM of 6 independent experiments. The average light units/µg protein from p21-5′ at 0.024% galactose was 2.1 million. Solid arrows indicate a ¼-binding site. (B) p53 functions from a ¾-site RE in the PIDD RE. Removal of the natural 8 bp spacer increased transactivation as expected; however, p53 was also able to transactivate from the natural PIDD RE containing the spacer. To determine the true functional p53 binding element, the canonical PIDD RE was broken into two noncanonical sites, ¾ PIDD-A and ¾ PIDD-B, which incorporated the spacer sequence into the RE and assessed for transactivation capacity with the luciferase assay. Solid arrows indicate ¼-binding site; dashed arrows indicate putative ¼-binding sites. (C) Analysis of p53 transactivation from various p53 targets in the genome that may be functioning ¾-sites. Shown are the identified p53 target sequences for p21-3′, PCNA, 14-3-3σ site 2 and APAF1 with the noncanonical ¾-site RE contained in these sites (identified by solid arrows).

We also addressed the ability of p53 to transactivate from ¾-site REs. A consensus (Con) binding site was created for each of the two possible configurations of a ¾-site RE. The first, designated Con J, consists of a ¼-site directly adjacent to a ½-site, whereas the second, designated Con K, contains a 5 base spacer between the ¼-site and the ½-site. Transactivation in the diploid yeast system from the Con J and Con K ¾-site REs was 25% and 18%, respectively, of the full p21-5′ RE level (Figure 5A), at high p53 expression (0.024% galactose). This was substantially more than observed for half-site REs. Similar findings were observed in the haploid yeast system (Figure S3A). Further evaluation of the sequence requirements for p53 transactivation from ¾-sites with the haploid yeast system showed p53 could transactivate from a ¾-site RE containing a CTTG core (Figure S3B). Transactivation was also effected by surrounding flanking sequences.

### Canonical REs Containing Spacers May Actually Be Noncanonical ¾-Site REs

In our examination of full-site RE sequences containing spacers, several REs contradicted our finding that large spacers between decamer half-sites abolish p53 transactivation. For example, the PIDD RE (p53 induced protein with death domain) [67] which promotes apoptosis has an 8 nt spacer in its RE. Contrary to our expectation, high expression of WT p53 resulted in ∼20% of the level of transactivation from the p21-5′ RE. As expected, removal of the 8 nt spacer between the decamer half-sites increased the levels of p53 transactivation (Figure 5B).

Examination of the PIDD RE sequence suggested that rather than functioning from a canonical full-site RE, p53 might transactivate from a noncanonical ¾-site RE. The canonical PIDD element was first separated into two noncanonical ¾-site REs (designated PIDD ¾ A and PIDD ¾ B), both of which utilized the “spacer” sequence as part of the binding element. The noncanonical REs were comprised of a ¼-site directly adjacent to a ½-site, but differed in the central CWWG motif in the half-site and number of mismatches from the consensus binding sequence. Transactivation assays revealed p53 could function from the PIDD ¾ A sequence to levels equivalent to the canonical PIDD RE containing the 8 nt spacer at high expression (Figure 5B). In contrast, WT p53 could not transactivate from the PIDD ¾ B RE. These findings showed that the PIDD RE was neither a true canonical consensus RE nor an exception to the “spacer” rule, but rather a noncanonical ¾-site RE which can support p53 transactivation.

The results of the PIDD RE sequence analysis suggested that other known p53 REs previously identified as canonical full-site REs are actually ¾-site REs. Based on an empirically derived set of RE rules previously established in our lab to predict p53 transactivation capacity [14],[30],[68], several established p53 target REs were re-examined to determine if their responsiveness to p53 was actually due to the presence of a noncanonical ¾-site RE instead of a full-site RE. As shown in Figure 5C, the REs of p21-3′, PCNA, 14-3-3σ (site 2) and Apaf-1 REs were actually comprised of a genuine ¾-site binding element (containing a ½-site directly adjacent to a ¼-site) followed by an additional sequence that vastly deviated from a consensus ¼-site. Luciferase assays in the diploid yeast strains revealed that p53 transactivation from these sites was clearly reduced in comparison to transactivation of p21-5′ RE (Figure 5C), but comparable to the levels of transactivation from the consensus ¾-site REs, Con J and Con K (see Figure 5A). These results are consistent with p53 transactivation capacities towards the p21-3′, PCNA, and Apaf-1 REs assessed with the haploid yeast system in a recent study focused on examining conservation of RE sequence and conservation of RE functionality [68].

### Structural Requirements for p53 Transactivation from ½- and ¾-Site REs

We examined several p53 mutations that could address the structural requirements for transactivation from noncanonical REs (½- and ¾-site REs) in comparison to canonical REs (weak and strong full-sites). The following REs were examined: ½-site REs (con G, con B, p21-5′ left, and p21-5′ right), ¾-site REs (con J and con K), as well as weak (TIGAR and Gadd45) and strong (p21-5′ and TIGAR-spacer) full-site REs. Mutations in the oligomerization domain were analyzed for transactivation capacity to determine the extent to which the tetramerization of p53 facilitated functionality from noncanonical REs. Also examined were N-terminal transactivation domain and C-terminal regulatory domain mutants. The results are summarized in Table 1 and presented relative to transactivation from the p21-5′ RE. The variation in transactivation by the p53 mutants towards the REs was not due to differences in levels of expressed p53 protein (Figure S4).

**Table 1:** Transactivation capacity of WT and mutant p53 towards canonical and nncanonical REs.

SYSTEM	p53 ALLELE:						RE
	tetramer	altered tetramer		dimer	monomer	terminal Δ	
	WT	R337C	R337H	L344A	L344P	Δ 368	
							**FULL RE:**
1n	+++		+++	+/−^a^	+/−^a^	+++	
2n	+++	+	+	+/−^b^	−	++	p21-5′
mammalian	+++	+	++	++^d^	−	++	
1n	+++					++	TIGAR
2n	+++	+	+	+/−	−	+++	no spacer
1n	+					+/−	TIGAR
2n	+	+/−	+/−	−	−	+/−	natural
1n	++					+	Gadd45
2n	++	+/−	+/−	−	−	+	
							**¾ RE:**
1n	++		+	+	−	+	Con J
2n	++	+/−	+/−	+/−	−	+	
1n	++		+	+	−	+/−	Con K
2n	+	+/−	+/−	+/−	−	+	
							**½ RE:**
1n	+/−			−	−	+/−	Con G
2n	+/−	−	+/−	−	−	+/−	
1n	+/−			−	−	−	Con B
2n	+/−	−	+/−	−	−	+/−	
mammalian	+ [y +/−]	−	+/−	+/−	−		p21-5′ left
mammalian	+ [y +/−]	−	+/−	+/−	−		p21-5′ right

WT p53 and several structural mutations were examined in three *in vivo* systems (haploid [1n] and diploid [2n] yeast and human SaOS2 cells) for transactivation from REs representing full, ¾-sites and ½-sites. The ability of the p53, WT or mutant, to transactivate from a RE was measured with a luciferase assay and compared to the ability of WT p53 to transactivate from the p21-5′ RE at 0.008% and 0.024% galactose in 1n and 2n yeast, respectively, or compared to the pGL3 plasmid lacking the promoter REs in human cells. Relative transactivation by a p53 variant in comparison to WT p53 towards the p21-5′ RE is represented by +++ (75–100%), ++ (25–75%), + (7.5–25%), +/− detectable but weak response, or − (no detectable response). The R337C and R337H mutations are compromised for their ability to form tetramers (the equilibrium between dimers and tetramers is altered in comparison to WT p53) [86]. The L344A and L344P mutations result in p53 protein being present as a dimer and monomer, respectively. The Δ368 mutation deletes the C-terminal basic domain required for structure-specific and non-sequence specific binding but has no effect on dimer or tetramer formation. “a” corresponds to significantly higher response at high galactose concentrations (0.128% and 2%). “b” indicates that the mutant was capable of transactivation from RE in an *ADE2* plate assay at high galactose concentrations. “d” indicates that the dimer mutation N345S was also analyzed in the mammalian system and yielded the same results as L344A.

The Arg337 residue is located on the surface of the tetramerization domain and plays a role in tetramer stabilization through a salt bridge with Asp352 on the opposite monomer [69]–[73]. The mutations R337C and R337H, associated with Li-Fraumeni-like syndrome (LFL) and adrenal cortical carcinoma (ACC), respectively, appear to compromise the ability to form tetramers. The impact of the R337C mutation (previously described as a partial function mutation) on p53 functionality has been postulated to arise by a change in thermodynamic stability, shifting the equilibrium from the tetramer to dimer and/or monomeric states [74],[75]. The R337H mutation is considered to have a subtle functional effect causing a pH dependent effect on folding [69], [74], [76]–[79] (Storici and Resnick, unpublished data).

In agreement with previous findings by Lomax et al.[74], R337C was found to be an altered function mutation in the diploid yeast and SaOS2 cell systems, significantly diminishing transactivation from both canonical and noncanonical REs. Transactivation from the strong, full-site REs was reduced to less than 25% of the levels of WT p53 (“+” in Table 1), whereas transactivation from the weaker full-sites and the ¾-site REs was barely detectable (“+/−” in Table 1) with no transactivation from the ½-site REs.

Similar to previous results [74], the p53 R337H mutant could transactivate from the p21-5′ RE to levels comparable to WT p53 in the haploid yeast system. However, although R337H could function from the p21-5′ RE in the diploid yeast and SaOS2 cell transactivation assays, the level was reduced in comparison to WT p53. Similar results were found for transactivation from the weaker full-site REs and ¾-site REs. The differences between WT and mutant p53 were not as clear for the ½-site REs.

L344 is one of five residues that comprise the hydrophobic core of the α-helices which form the interface for p53 dimer-dimer interactions [70],[72],[73],[80],[81]. The mutation L344A prevents tetramer formation although stable dimers are formed [72],[80],[82],[83]. The missense mutation L344P disrupts the helix [74], [84]–[86] and is associated with Li-Fraumeni syndrome (LFS). This alteration prevents dimer formation resulting in monomeric p53 protein [74], [75], [86]–[89]. Within the haploid and diploid yeast systems, the L344A protein had reduced transactivation activity towards the strong canonical full-site REs and noncanonical ¾-site REs (Table 1) and no activity towards half-sites. However, the L344A protein was capable of transactivating to low levels from the p21-5′ half-site REs within mammalian cells. Similar results were seen in SaOS2 cells with an N345S dimer mutation, where transactivation was measurable, but significantly reduced in comparison to WT p53 transactivation (data not shown).

Consistent with previous findings showing L344P is a loss-of-binding mutation, the monomeric p53 L344P was not able to transactivate from the full-site, ¾-site, or ½-site REs tested within the 2n yeast or SaOS2 cells (Table 1) [74], [75], [86]–[89]. Similarly, the germline mutation L330H, which is predicted to form only monomers through destabilizing the β-strand of the tetramerization domain [80],[87],[90], was also inactive for transactivation from the p21-5′ full-site RE and half-site REs in the mammalian system (data not shown).

Finally, deletion of the tetramerization domain (Δ325–357) or a terminal truncation (331 stop: similar to the p53β isoform) resulted in complete loss of transactivation from any of the binding sequences examined, including full-site REs, in the yeast or SaOS2 cell systems (data not shown). These results were in agreement with previous findings that the tetramerization region is necessary for p53 binding to and transactivation from a RE [83],[91].

Previously, we reported that the terminal deletion Δ368 resulted in reduced transactivation capacity towards full-site REs when assessed with a yeast *ADE2* reporter plate assay (on plates) in haploid yeast [30]. Those studies have been extended to address its ability to transactivate from full-, ¾- and ½-site REs. We found that removal of the C-terminal tail resulted in at most a modest transactivation from the noncanonical and canonical REs (Table 1). The inhibition varied with the strength of the RE sequence, as well as with level of p53 expression (data not shown) for all REs. Thus, in comparing WT to mutant, the C-terminal domain of p53 appears in several cases to actually enhance rather than repress transactivation from canonical, as well as noncanonical REs.

Finally, we also examined a deletion in the N-terminal domain, Δ1–39, to assess if transactivation from ¾- and ½-site REs was differentially affected. The mutation resulted in <25% residual transactivation from the full-site REs (strong: p21-5′ and TIGAR - spacer; moderate: GADD45) at low expression in the haploid yeast system (data not shown). At the high expression levels required to examine noncanonical REs (0.128% galactose), transactivation was also reduced from ¾-site REs (con J and con K) and a ½-site RE (con G) suggesting that the canonical and noncanonical REs are similarly affected by defects in the transactivation domain.

## Discussion

Given the role for p53 in assuring genome stability, it is important to understand how this master regulator functions as a sequence-specific transcription factor. The interactions of p53 bound to DNA, as well as the mechanisms by which WT or mutant p53 can transactivate to different extents from the many variants of the consensus motif are not completely understood. In this work, we have developed a matrix of factors influencing transactivation that include WT and mutants, sequence and motif, and DNA binding. We have established that p53 can function from many noncanonical ½- and ¾-site REs that are common to the genome. Importantly, functionality of a sequence is not directly predictable by relation to the consensus sequence.

### Isogenomic System To Address p53 Transactivation from Many REs in Diploid Yeast

Yeast has been extensively used as an *in vivo* test tube to analyze p53 transactivation capacities towards p53 response elements (REs) derived from human genes and assess the potential role that target sequence plays in p53 functionality [3],[14]. A distinct advantage of yeast is the opportunity to rapidly modify *in vivo* either the target REs or to create mutant p53 coding sequences utilizing a highly efficient recombination-based system, known as *delitto-perfetto* [Italian for “perfect murder”] that targets desired changes with oligonucleotides [49],[92],[93]. The newly developed diploid yeast approach extends our previous work with a plasmid-based haploid yeast system in order to capitalize upon the opportunity to conveniently merge a large number of integrated (single copy) p53 mutants with transactivation capabilities at many response elements simply through mating of strains. Not only can the rheostatable, diploid yeast system differentiate between functional and nonfunctional REs, it can also estimate weak to strong functionality at different levels of p53 expression. The results in yeast have proven useful in guiding studies with highly expressed WT and mutant p53 towards potential target REs in human cells and evaluations of direct DNA binding.

### Spacers Can Have Opposite Effects on Transactivation

Previous studies have shown that widely-separated full-size REs associated with the same p53 target gene, such as the muscle creatine kinase (MCK), can interact to synergistically transactivate the associated target gene [94]. The mechanism has been suggested to involve looping out of the intervening DNA so that multiple p53 tetramers can “stack” and concentrate the basal transcription machinery [94],[95]. While this mechanism of transactivation may hold for REs separated up to 3 kb, the synergy was proposed to be lost when the distance was less than 25 bases due to steric hindrance [94], an arrangement that holds for several p53 target genes including MDM2. We found that p53 could function synergistically from the two weak MDM2 REs separated by a 17 and 10 nt spacer, but further reduction to 5 nt resulted in additivity.

Interestingly, the length of the spacer between the two REs within the promoter of *Mdm2* has been conserved between mouse and human although the sequence of the spacer is 50% diverged. In the haploid yeast system, p53 was found to function similarly from the human and murine MDM2 REs in terms of synergistic transactivation [68]. Given the divergence in the sequence of the spacer, it is unlikely that an additional transcription factor binding site would be found in the 17 nucleotides that would allow a second transcription factor to interact cooperatively with p53 to induce the observed synergistic transactivation from the two REs. Instead, there may be a functional conservation that assures the level of p53-mediated transactivation. This is supported by sequence analysis across 14 species, corresponding to at least 70 million years of evolution, where 12 had maintained the 17 nucleotide spacer (with varying degrees of sequence divergence) between RE1 and RE2 (including chimp, rat, rabbit, dog, elephant, armadillo and hedgehog), while opossum and bat had a one nucleotide indel [96],[97]. From this preliminary search, it appears that the spacer sequence is under stabilizing selection such that variations in the length of the spacer which would affect the ability of p53 to synergistically function from these sites were not observed. Interestingly, a similar phenomenon, is observed in the DNA sequences within and between (but not flanking) cis-regulatory elements of the *otx*, *delta*, *wnt8*, and *brachyury* genes where insertions or deletions of random sequence do not occur as assessed by a comparison between the orthologous cis-regulatory regions and flanking sequences of the sea urchins, *Strongylocentrotus purpuratus* and *Lytechinus variegatus* [98]. We are currently investigating whether functional conservation of spacer length as a mechanism for regulating p53 functionality holds with other closely-spaced full-sized p53 REs.

The impact of the spacer between full-sites acts opposite to that of a spacer between half-sites. In previous studies, it was proposed that REs had a “rotational specificity” where spacers between half-sites could abrogate p53-dependent transactivation if they were on opposing faces of the DNA helix, but had little impact on transactivation when the half-sites were on the same face [18],[59],[60]. Recently, in electrophoretic mobility shift binding assays (EMSA), a 2 nt spacer did not significantly alter p53 affinity (K_d_) towards a consensus RE [29]. Furthermore, in a 3D model derived through cryoelectron microscopy of full length p53, where p53 dimers are proposed to form through interactions of α-helices on the N- and C-termini rather than α-helices on the C-termini, the best fit RE sequence had a spacer length of 6 nucleotides [99]. These *in vitro* findings imply if p53 transactivation is primarily dependent on the ability of p53 to bind target REs, a small spacer would not affect p53-dependent transactivation.

The effect of spacer upon transactivation from a RE at varying levels of p53 was first analyzed with the WAF1/Cip1 p21-5′ RE. The present results are in agreement with an earlier report showing that increases in spacer length of 4 or 14 bases between half-sites decreases p53 transactivation in yeast [18]. However, there is no evidence for rotational specificity influencing transactivation. This is the first demonstration that a spacer of 1 or 2 nt between half-sites can dramatically affect the ability of p53 to transactivate from a RE. Importantly, the impact of spacer on the ability of p53 to transactivate from a RE is greatly influenced by the intrinsic potential transactivation strength of the RE, as shown for NOXA, as well as level of p53 expression. We also established with the haploid yeast system that a spacer had a similar negative effect on the ability of p53 family members, p63β and p73β, to transactivate from a RE.

The biological importance of spacer as a means for affecting response to p53 was clearly demonstrated for the TIGAR RE. We propose that unlike the implications from the established consensus RE, a spacer between half-sites in natural elements serve to modulate the levels to which p53 can regulate the associated target gene and may play an important mechanistic role in the evolution of RE responsiveness to p53. In the case of TIGAR, the opportunity to address evolutionary implications is limited due to the apparent recent emergence of the putative p53 binding site in the primate lineage, however, the orthologous TIGAR RE in *Pan troglodytes* (chimpanzee), and *Macaca mulatta* (rhesus monkey) also contain a 2 nt spacer [96],[100].

### Binding vs. Transactivation

Several modes of binding have been postulated for p53 to a RE containing a spacer that include shifting of the tetramer on the DNA to compensate for the reduced protein-protein interactions, bending and/or kinking of the DNA within the spacer and/or RE and rotational motions of p53 to accommodate supercoiling of the spacer sequence [24],[27]. In any of these, binding would modify either the conformation of the p53 tetramer and/or the DNA sequence itself in a fashion that is not predictable from current structural studies. While functional studies demonstrate that the RE sequence can dramatically influence the level to which p53 can transactivate from a specific RE [3], the relationship between *in vitro* binding and efficiency of *in vivo* transactivation had not been established prior to this study.

We utilized a novel fluorescent microsphere, semi-*in vitro* binding assay to determine the effect of spacer upon p53 binding. Recently, we demonstrated a good correspondence between binding and *in vivo* transactivation for several human p53 target REs (Noureddine et al., submitted). While differences were observed between the *in vivo* and *in vitro* assays for p53 binding to the p21-5′ RE containing a single nucleotide spacer, increasing the length of a spacer between the decamer half-sites beyond one nucleotide had a large impact on the ability of p53 to bind in the semi-*in vitro* assays, contrary to reports with pure components [22],[23]. Interestingly, p53 binding to a RE with a ≥3 nt spacer was nearly equivalent to binding to the individual p21-5′ half-site REs, suggesting that p53 may recognize each decamer sequence as a distinct binding motif once a spacer increases beyond a certain length. These findings strongly argue that the 0–13 nucleotide spacer in the established p53 consensus sequence, which is based on interactions between purified protein and REs, should be reduced to no more than a few bases. It appears that factors present in nuclear extracts or cells limit the ability of p53 to recognize REs with spacers between half sites. It will be interesting to identify these putative spacer-discriminating factor(s).

### p53 Functionality from ½-Site and ¾-Site REs

The observations that p53 can function, as defined by binding and transactivation, from consensus half-site REs in yeast and from the p21-5′ half-site REs in mammalian cells indicate the p53 transcriptional network is comprised of many more downstream targets than previously predicted. Transactivation from half-sites is strongly dependent upon the targeted sequence and level of p53 expression. Similar to full-site REs [30], the central binding motif at the monomer junction (*i.e.*, CATG) had the greatest impact upon the ability of p53 to function from half-sites. The difference between the yeast and mammalian systems in functionality from the half-sites indicates that additional co-factors in human cells may assist in p53 interactions with weaker binding sequences. Tetrameric p53 can bind full-site and half-site binding elements *in vitro*, but the p53 bound to a half-site had a much higher disassociation rate *in vitro* as indicated through “trap” assays that had measured dissociation of p53 from a labeled RE sequence [22].

Given the higher probability of a decamer sequence occurring in the genome compared to a 20 base sequence, it will be interesting to determine the number of functional p53 half-site REs in the human genome which is composed of ∼3 billion base pairs. In a preliminary genomics screen, we have identified over 1,400 “perfect” p53 half-sites containing a CATG core motif in the genome within 2 kb of a transcriptional start site. However, the question remains as to which of these sites will have a relevant biological function in p53-dependent stress responses or whether the sequences are merely “noise” within the genome.

Half-sites could serve many roles in the genome. For example, a collection of half-sites could affect chromatin accessibility to transcriptional machinery (*i.e.*, loosening the chromatin or recruiting and sequestering chromatin modifiers). Such sites might function in the proposed opening of the genome to make it more available for repair [101]. On the other hand, half-sites might also serve to titrate p53. Additionally, p53 half-sites may play a role in bringing together different transcriptional networks as described for a SNP in the *FLT-1* promoter [41],[66]. Subsequent studies of the *FLT-1* half-site have shown that p53 bound to the T-SNP can cooperate with estrogen receptor (ER), also bound to a half-site of its own consensus sequence, to synergistically transactivate from the *FLT-1* T promoter [66]. Such co-regulation may be advantageous for precise fine-tuning of p53-regulated responses and may make activity from the noncanonical ½-site REs more dependent on availability of cooperating transcription factors or additional cofactors and on levels of nuclear p53.

Within yeast, p53 was able to transactivate from noncanonical consensus ¾-site REs containing a ¼-site adjacent to a ½-site or a ¼-site separated from a ½-site by a 5 nt spacer. The lack of a significant difference between the transactivation from both ¾-site REs imply that p53 encounters the ¾-site RE as a tetramer protein. Transactivation from ¾-site REs was moderate relative to full-sites. Furthermore, the hierarchy of transactivation, in terms of the level to which p53 could bind and transactivate *in vivo*, was comparable to earlier *in vitro* studies that measured p53 binding efficiencies towards ½-sites, ¾-sites and full-site sequences with competitive gel retardation assays [59]. The impact of a ¾-site was revealed in our study of the RE for the PIDD gene where p53 transactivation was substantial even though the RE contained a spacer greater than 3 nt. Transactivation assays revealed the responsiveness of the originally described PIDD RE was actually due to a noncanonical ¾-site RE. Transactivation from the PIDD ¾ A RE, but not the PIDD ¾ B reinforces the requirement for a strong core element for functionality and that a functional RE cannot contain greater than 3 mismatches within a half-site. There are likely to be many functional ¾-site REs in the genome, among which are sequences that were originally considered as canonical, full-site REs containing >2 nt spacers. Furthermore, as recently pointed out in our functional conservation analysis of p53 REs from several species, there are clear examples of weaker, apparently noncanonical ¾-site REs, such as p21-3′ and APAF1 being conserved in evolution [68].

While this report is the first to systematically evaluate p53 function from noncanonical ½- and ¾-site REs, there are other reports of a transcription factor binding to noncanonical binding sites within the genome. Johnson *et al.* [102], recently mapped the *in vivo* interactome for the transcription factor neuron-restrictor silencing factor (NRSF) using a large scale ChIP analysis and found that NRSF bound to noncanonical half-site binding motifs. A variety of approaches such as ChIPSeq or FAIRE, formaldehyde-assisted isolation of regulatory elements, may be able to reveal interactions at noncanonical sites under different stress conditions and in various cell types [103],[104] to better understand the dimensions of the p53 master regulatory universe.

### Transactivation From ½-Site and ¾-Site REs Require Tetrameric p53

Having established WT p53 interacts with sequences that do not fully conform to the canonical p53 binding sequence, a panel of p53 mutants was analyzed to determine what structural features of p53 play a role in transactivation from ½- and ¾-site REs versus full-site REs. Together, the mutations suggest that common functional aspects of p53 are required for the ability of p53 to function from full, ¾-, and ½-site REs. In general, the observed transactivation of oligomerization mutations towards full-site REs was in agreement with previous reports of mutants able to form tetramers or dimers retaining transactivation towards strong REs, while monomeric proteins were inactive [89].

Having established WT p53 interacts with noncanonical sequences, a panel of p53 mutations was analyzed to determine what structural features of p53 play a role in transactivation from ½-site and ¾-site REs versus full-site REs. The observed transactivation of oligomerization mutations towards full-site REs has confirmed previous reports of mutants able to form tetramers or dimers retaining transactivation towards strong REs, while monomeric proteins were inactive [89]. Together, the mutations suggest that common functional aspects of p53 are required for the ability of p53 to function from full, ¾-, and ½-site REs.

The DNA binding domain of p53 has been shown *in vitro* to bind DNA in the absence of the oligomerization domain and that dimeric p53 can bind to half-sites in a cooperative fashion independent of tetramerization [22],[105]. While able to transactivate at a low level from strong full-site REs and ¾-site REs, the L344A dimerization mutation was unable to transactivate from the weak full-site REs or consensus half-site REs in yeast. In agreement with these findings, Waterman *et al.* previously showed that the L344A protein could bind *in vitro* to oligonucleotides containing an optimal consensus binding site and/or half-site, but not to an oligonucleotide containing a suboptimal full-site [83].

The impact of the dimer mutation L344A, as well as N345S, was less severe in mammalian cells. Similarly, a designed dimer mutant (Met340Gln/Leu344Arg) was capable of transactivating from a p21 full-size RE within SaOS2 cells to half the level of WT p53 suggesting a direct correlation between the oligomeric state and transactivation activity [75]. While L344A dimeric proteins may bind to the sites in a cooperative fashion, tetramerization which is a necessity for efficient transactivation may be required to stabilize the binding and reduce the off-rate of p53 from the DNA in yeast. It is possible that other factors in SaOS2 cells may contribute to this stabilization accounting for the difference between the systems.

Since altered tetramer proteins and dimers exhibited a reduced ability to transactivate from noncanonical REs, transactivation is not simply an additive process determined by the number of available pentamer sequences. It is possible that binding and transactivation *in vivo*, but not binding alone, may require a conformational change of the p53 protein that is only obtainable with a tetrameric protein. Several transcription factors have displayed such a characteristic including the heat shock protein which can bind chromatin weakly as monomer, but is not sufficiently active to cause a biological response until after it is induced and forms a trimer [106],[107]. Furthermore, in the case of a half-site, where there is not a sequence to which the second p53 dimer can bind, tetramerization may assure the second dimer is in the vicinity of the p53 binding element in order to interact with other factors required for transactivation.

The finding that the p53 Δ368 mutation reduced transactivation from both canonical and noncanonical REs agrees with studies showing that p53 does not require modification of the C-terminal to engage its target binding sites [31],[32]. Furthermore, these results indicate that the C-terminal is not absolutely essential for function from canonical or noncanonical sites, but may play a role in regulating the level of transactivation.

The deletion of the first N-terminal transactivation domain (TAD) which mimics a naturally occurring alternatively spliced form of p53 that is differentially expressed in breast tumors, had a more severe impact upon transactivation than previously reported with the qualitative *ADE2* color reporter (data not shown) [30],[108]. The results may reflect the greater quantitative assessment of transactivation with the luciferase assay. Nevertheless, both assays revealed that the decreased opportunity to interact with the transcriptional machinery through the loss of the transactivation domain strongly affects p53 transactivation from both canonical and noncanonical REs.

### Conclusions and Implications

Through deconstructing the canonical consensus sequence and assessing functionality, as determined by binding and transactivation within three *in vivo* model systems and a semi-*in vitro* binding assay, it has been possible to refine the requirements for functional p53 binding elements. The organization and arrangement of binding motifs, as well as the level of p53 expression, have a large impact on the ability of p53 to transactivate from a RE sequence. It is interesting that p53 may not function from reported canonical consensus REs yet functions from noncanonical REs that are common to the human genome. Half-sites and spacers between full-sites may provide additional levels of regulation in p53 transcriptional network. Truly functional REs may be restricted to decamers separated by <3 nucleotides. When the spacer increases beyond 3 nucleotides, we suggest p53 recognizes the half-sites as separate binding entities.

The ability of p53 to function from noncanonical decamer half-sites introduces a new realm of target sequences and genes into the p53 transcriptional network and expands the universe of p53 regulated genes. Noncanonical sequences might provide p53 responsiveness at high levels of expression or in combination with other transcription factors, as for the case of the *FLT-1* gene [66]. As discussed in the “piano model” for transactivation from a broad range of REs [14], variation in the level of p53, as well as various mutants can markedly affect the spectrum of p53 responses. Determining the relationship between expression levels and responsiveness at canonical and noncanonical target sequences is important in understanding how p53 implements cellular fate in response to stress, such as cell cycle arrest or apoptosis. It is also important for addressing the consequences of p53 alterations, particularly those mutations that retain transactivation capabilities, as well tailoring individual therapies.

## Materials and Methods

### Construction of Isogenomic Diploid Yeast Strains Containing p53 Inducible Reporters

The site-directed mutagenesis system, *delitto perfetto* [48],[49] was used to generate a panel of isogenic “p53-host” strains and a panel of response element (RE) reporter strains in the budding yeast, *S. cerevisiae*. Each “p53-host” strain, yAT-iGAL::p53 (*MAT* ***a*** *leu2-3,112 trpl-1 his3-11,15 can 1-100 ura3-1*, *trp5::pGAL1:p53:cyc1-Ter*, *lys2::Hygro^R^*), contains the wild type p53 cDNA controlled by the inducible, “rheostatable” *GAL1* promoter [109] integrated at the *TRP5* locus on chromosome VII. p53 mutations in the tetramerization and basic domains were constructed using a derivative of the p53 host strain containing a CORE cassette (CO, counterselectable, KL*URA3*; RE, reporter, KanMX4 resistance gene) integrated within the p53 cDNA at nucleotide position 1105 [110]. Modification of the p53 cDNA was performed using the *delitto perfetto* approach [*48*],[49**] where the CORE cassette was replaced with an oligonucleotide containing the mutation of interest to generate a full-length mutant p53 cDNA or the desired deletion of the C-terminus. Replacement of the CORE was confirmed by selection on 5-FOA and kanamycin sensitivity. Specific p53 alterations were confirmed by colony PCR and sequencing (Big dye, Applied Biosystems, Foster City, CA).

The second panel of isogenic strains containing p53 REs upstream of the *CYCl* minimal promoter and the firefly luciferase reporter were obtained starting from the yLFM-ICORE strain, as previously described (Table S1) [42]. The reporter strains are also isogenic with the p53 host strains, but *LYS2* and Hygro^s^.

Mating of the reporter and p53-host strains followed by selection for diploid cells on Lys− Hygro+ plates, results in *isogenomic* yeast that enable the assessment of the transactivation potential for WT or mutant p53 proteins towards individual REs in the p53 transcriptional network. Strains differ only by the mutation of interest and 4–5 nucleotide variation in the RE.

### Quantitative Luciferase Assay in Diploid Yeast

Individual p53-inducible RE reporter colonies were inoculated into 5 ml rich media, YPDA plus adenine [200 mg/L], and grown overnight at 30°C with shaking. The overnight culture was diluted 1∶50 in H_2_O. For each reaction, 1 ml of the diluted culture was spun down, washed of residual glucose with H_2_O and re-suspended in 2 mL synthetic complete - LYS media containing 2% raffinose or raffinose supplemented with either 0.008% or 0.024% galactose. These dilute raffinose cultures were grown overnight (∼18 hr) at 30°C to ∼2–4×10^7^ per ml. The 2 ml cultures were spun down and the supernatant was aspirated. The remaining pellet was resuspended in 100 µl reporter lysis buffer (Promega, Madison, WI) and an equivalent amount of 425–600 micron acid-washed, glass beads was added (Sigma, St. Louis, MO). Samples were homogenized for 30 seconds in the Biospec Products, Inc. mini-bead beater (Bartlesville, OK), briefly incubated on ice and spun for 20 minutes at 16k relative centrifugal force (rcf) in an Eppendorf 5415R centrifuge (Batavia, IL) to separate the soluble protein fraction. The standard protocol recommended by the manufacturer (Promega; Madison, WI) was performed for the luciferase assay system starting with 10 µl of protein extract. Luciferase activity was measured from 96-well, white optiplates (Perkin Elmer, Waltham, MA) in a Wallac Victor^2^ multilabel counter (Perkin Elmer, Waltham, MA). Light units were standardized per µg protein as determined by a Bio-Rad protein assay (Bio-Rad; Hercules, CA). Luciferase assays in haploid strains were performed as previously reported [14].

### Human Cell Lines

Human SaOS2 (HTB-85, ATCC) osteosarcoma cells were grown in McCoy's A5 medium supplemented with 10% FBS and 1× penicillin/streptomycin (Gibco, Carlsbad, CA). All cultures were incubated at 37°C with 5% CO_2_. Human lymphoblastoid cells were grown in RPMI 1640 media supplemented with 15% heat-inactivated fetal bovine serum (Invitrogen, Carlsbad, CA) and incubated at 5% CO_2_ at 37°C with 1% penicillin-streptomycin antibiotics (Invitrogen). The lymphoblast cell lines GM12824 and GM12825 used in the semi-*in vitro* binding assay were purchased from Coriell Cell Repositories (Camden, NJ).

### Transfections and Luciferase Assays in Human Cell Lines

Plasmids pC53-SN3 [111] coding for human p53 cDNA under the control of CMV promoter and the control vector pCMV-Neo-Bam were kindly provided by Dr. Bert Vogelstein. p53 mutations within the pC53-SN3 vector were generated by site-directed mutagenesis (QuickChange site-directed mutagenesis kit, Stratagene) and were confirmed by sequencing. Luciferase reporter constructs containing the p53 REs were constructed in pGL3-Promoter backbone (Promega, Madison, WI). Partially complementary oligonucleotides containing the RE and 5 additional flanking nucleotides from both sides were annealed *in vitro* to yield double-stranded molecules that would be compatible for an *in vitro* ligation reaction with XhoI/BamHI double digested pGL3-promoter vector. Ligation products were transformed into XL1 blue *E. coli* cells, purified, amplified and sequenced. pRL-SV40, a reporter plasmid coding for *Renilla reniformis* luciferase (Promega, Madison, WI) was used as a control of transfection efficiency in the luciferase reporter assay.

For luciferase assays SaOS2 cells were seeded in 24-well plates 24 hours before transfection. Cells were transfected using Fugene-6 (Roche, Indianapolis, IN) according to manufacturer's instructions at ∼80% confluence (with 200 ng of reporter constructs). When appropriate, 25 ng of the p53 was co-transfected. Total plasmid DNA per well was adjusted to an equal level by adding the empty vector pCMV-Neo-Bam. Forty-eight hours post transfection, extracts were prepared using the Dual Luciferase Assay System (Promega) following the manufacturer's protocol and luciferase activity was measured in a Victor Wallac multilabel plate reader (PerkinElmer). For each construct, relative luciferase activity is defined as the mean value of the firefly luciferase/Renilla luciferase ratios obtained from at least three independent experiments.

### Chromatin Immunoprecipitation (ChIP) Assays

SaOS2 cells were seeded in 10-cm dishes and transfected at 80% confluence with WT p53 expression vector along with the pGL3-P reporter plasmid containing the p21-5′ RE or its derivatives. ChIP on plasmid assays were performed as described previously [66] using the ChIP kit (Upstate Biotechnology) following the manufacturer's instructions. A mouse monoclonal anti-p53 antibody DO7 (Pharmigen, BD) was used. PCR amplifications were performed on immunoprecipitated chromatin using a pair of primers to amplifya specific region in the pGL3-P backbone containing the p53 REs (5′-ATAGGCTGTCCCCAGTGCAA-3′ and 5′-TGGAATAGCTCAGAGGCCGA-3′). The PCR cycles were as follows: an initial 10 min Taq Gold polymerase at 95°C followed by 40 cycles of 95°C for 15 s and 60°C for 1 min. The PCR products were then run on a 1.8% agarose gel and quantified with IMAGEQUANT V5.1 (Molecular Dynamics-GE, Piscataway, NJ).

### Semi-*in vitro* Binding Assay with Human Nuclear Extracts

To evaluate the sequence-specific p53-DNA binding interactions, we used the semi-*in vitro* fluorescent microsphere binding assay as previously described (Noureddine et al., submitted). Briefly, lymphoblast cells were grown to ∼8.5×10^5^ cells/mL before exposing to 0.6 ug/mL (1 mM) doxorubicin (Sigma, St. Louis, MO) for 18 hours at 37°C. Nuclear protein was extracted from non-treated and treated cells using a Nuclear Extraction Kit according to manufacturer's protocol (Active Motif, Carlsbad, CA) and protein concentration was measured in triplicate using the BCA Protein Assay Kit (Pierce, Rockford, IL), followed by a plate read using a Perkin Elmer HTS 7000 BioAssay Reader. All the oligonucleotides reported in this study were synthesized by Invitrogen (Carlsbad, CA). Fluorescent microspheres bearing double stranded DNA fragments containing a sequence of interest were multiplexed and incubated for 1 hour in the presence of 1.75 µg of nuclear protein extracts from either treated or non-treated cells. Following incubation in cell extracts, the beads were incubated with p53 antibodies (DO-7, BD Biosciences, San Jose, CA) and secondary antibodies conjugated with phycoerythrin(R-phycoerythrin-coated goat anti-mouse) for 30 minutes. The p53 interaction with each bead was measured on a BioPlex Machine (BioRad) as raw fluorescence intensity signal generated from phycoerythrin-conjugated secondary antibody to mouse anti-p53. For signal normalization, an aliquot of the DNA-conjugated beads was treated separately with phycoerythrin-conjugated streptavidin for 20 minutes in the absence of any nuclear extracts. All binding reactions were conducted in triplicate. For each bead type in every multiplex set of beads, relative binding signal was obtained by normalizing the absolute binding signals of extract-treated beads (mean of 3 replicates) to mean signals obtained from the same set of beads that were independently treated with phycoerythrin-conjugated streptavidin (mean of 3 replicates). This normalization accounts for bead type-specific oligo content. Net binding for each oligo is the numerical difference between NT and DOX-treated signal obtained for this oligo.

### Western Analysis

#### Yeast Cells

Individual yAT×yLFM diploid colonies were inoculated into YPDA media and grown overnight. Overnight cultures were diluted 1∶50 in H_2_O and 1 ml of the culture was spun down for each sample treatment, washed with 2 ml of H_2_O and inoculated into 2 ml synthetic media (lys-) with either glucose (2%), raffinose (2%) or raffinose plus increasing amounts of galactose (0.002–0.032% galactose). Cultures were grown 24 hours at 30°C with vigourous shaking. Protein extracts were prepared in 35 µl reporter lysis buffer (Promega, Madison, WI) plus 2% protease inhibitors (cocktail for use with fungal and yeast extracts; Sigma, St. Louis, MO). An equivalent amount of 425–600 micron acid-washed, glass beads (Sigma, St. Louis, MO) was added prior to the samples being homogenized for 30 seconds in the Biospec Products, Inc. mini-bead beater (Bartlesville, OK). Following homogenization, samples were briefly incubated on ice and spun for 20 minutes at 16k rcf in an Eppendorf 5415R centrifuge (Batavia, IL) to purify soluble proteins. Protein concentrations were measured with the Bio-Rad protein assay according to the standard protocol (Bio-Rad; Hercules, CA). 50 µg of total protein extracts were run on SDS-PAGE gels and transferred as previously described [109]. p53 was detected with the mouse monoclonal antibodies DO-1 and pAb1802 (Santa Cruz Biotechnology, Inc., Santa Cruz, CA) according to the manufacturer's protocol. GAPDH was detected with the rabbit polyclonal GAPDH antibody (HRP) (Abcam, Cambridge, MA).

#### Human Cells

Whole cell extracts from SaOS2 transfected cells were obtained using cell culture lysis reagent (Promega) following the manufacturer's instructions. Equal amounts of whole cell extracts were separated on 4–12% BisTris NuPAGE and transferred to polyvinylidene difluoride membranes (Invitrogen). The blots were probed with primary antibodies (Santa Cruz) for p53 (pAb1801 and DO-1) and Actin (C-11). Bands were detected using horseradish peroxide-conjugated secondary antibodies (Santa Cruz) and the enhanced chemiluminescence (ECL) detection system (Amersham, Cleveland, OH, USA).

## Supporting Information

Figure S1 Low level expression of p53 under the rheostatable GAL1 promoter. The GAL1 promoter allows for increased expression of p53 depending on the level of galactose in the media. With glucose supplemented in the media, the GAL1 promoter is repressed. Raffinose provides a basal level of expression where the promoter is derepressed, but not induced. Presented is a Western blot analysis of p53 expression 24 hours post-inoculation with 2% glucose (G), 2% raffinose (R) or 2% raffinose plus increasing amounts of galactose (0.002–0.008%). The p53 protein was detected with DO-1 and pAb1801 antibodies. Immunodetection with GAPDH was used as a standard loading control. To determine the relative protein concentrations at repressed, basal and low galactose levels, pixel values were measured from autoradiographs of increasing exposure lengths (from 1 to 15 minutes). The ratio of measured pixel values were calculated between samples and compared between exposure lengths to derive the relative differences in p53 expression between media containing glucose (2%), raffinose (2%) or raffinose (2%) plus increasing concentrations of galactose (data not shown). A 3-fold induction of p53 expression was observed between samples containing glucose and raffinose, as well as between raffinose and 0.004% galactose. There was a 2-fold induction between the 0.004% galactose and 0.006% galactose samples and a 3-fold difference between the 0.006% and 0.008% galactose samples. This totaled a relative increase in p53 expression of 54-fold between repressed conditions and induction at 0.008% galactose and an 18-fold between basal level expression and induction at 0.008% galactose.
(0.48 MB DOC)

Figure S2 Increase in spacer decreases transactivation by p53 family members. (A) The plasmid-based haploid yeast system [30] was utilized to determine the extent to which p53 transactivation was impacted by a spacer between decamer half-sites. Transactivation was measured as relative light units/µg protein at 0.008% and 0.128% galactose and compared to WT p53 transactivation from the p21-5′ RE. Depicted are the average light units/µg protein and standard deviations from 3 biological repeats. The average light units/µg protein for WT p53 towards p21-5′ at 0.128% galactose was 2.9 million. (B) The transactivation capacity of p63β and p73β from the p21-5′ RE containing spacers of 0, 2, 5 and 10 nt was measured to determine the affect of spacers on transactivation by p53 family members. Transactivation was measured at moderate and high levels of p53 expression.
(0.43 MB TIF)

Figure S3 Transactivation from noncanonical 3/4-site REs in haploid yeast. (A) The ability of p53 to transactivate from the consensus 3/4-site REs, Con J and Con K were determined in the haploid yeast system, as described in Inga et al. [30]. The ability of WT p53 to transactivate from the 3/4-site REs was measured 24 hours post inoculation into the galactose-supplemented media by a luciferase assay. The ability of WT p53 to transactivate from each 3/4-site RE was compared to the ability of WT p53 to transactivate from the p21-5′ RE at 0.128% galactose. Transactivation is depicted as the light units/µg protein and presented as the mean and standard deviation of 3 independent experiments. The average light units/µg protein from p21-5′ at 0.128% galactose was 2.9 million. Solid arrows indicate a 1/4-binding site. (B) Sequence dependence of p53 transactivation from 3/4-site REs. The plasmid-based haploid yeast system [30] was utilized to determine the sequence requirements for transactivation from noncanonical 3/4-site REs at high expression levels (0.128% galactose). The central core “CWWG” motif was altered from CATG to CTTG and flanking regions were changed as described. Transactivation from 3/4-site REs was measured with a luciferase assay and calculated as light units/µg protein.
(0.51 MB TIF)

Figure S4 Mutant p53 expression. To exclude the possibility that variation in transactivation capacity towards the canonical and noncanonical REs by mutant p53 was due to variable protein levels, the relative expression of p53 mutants was compared to WT p53. Western analysis was performed 24 hours following transient transfection of mock, WT p53 or mutant p53 pCMV plasmid vectors into SaOS2 cells. Thirty-five µg of total protein was run on 4–12% BisTris NuPAGE as described in the Materials and Methods. Staining of the gel with SimplyBlue SafeStain (Invitrogen) for total loading protein was performed to discriminate possible overlap of protein between the loading control actin and the truncated p53 protein Q331stop which run at comparable distances.
(2.98 MB TIF)

Table S1 Response element sequences.
(0.05 MB DOC)
