# Correction: Noncanonical DNA Motifs as Transactivation Targets by Wild Type and Mutant p53

**DOI:** 10.1371/annotation/13bc83be-2345-401d-b953-f1886e9fbdff

**Published:** 2008-07-01

**Authors:** Jennifer J. Jordan, Daniel Menendez, Alberto Inga, Maher Noureddine, Douglas A. Bell, Michael A. Resnick

The fourth and fifth authors' names were incorrectly listed. The correct names are: Maher Noureddine and Douglas A. Bell. The correct citation is: Jordan JJ, Menendez D, Inga A, Noureddine M, Bell DA, et al. (2008) Noncanonical DNA Motifs as Transactivation Targets by Wild Type and Mutant p53. PLoS Genet 4(6): e1000104. doi:10.1371/journal.pgen.1000104

