# Correction: Noncanonical DNA Motifs as Transactivation Targets by Wild Type and Mutant p53

**DOI:** 10.1371/annotation/f7fc9c28-14ae-480d-a69e-ee9cc4fba9a7

**Published:** 2008-10-20

**Authors:** Jennifer J. Jordan, Daniel Menendez, Alberto Inga, Maher Nourredine, Douglas Bell, Michael A. Resnick

The affiliation for the third author Alberto Inga was incorrect. Dr. Inga is only affiliated with institution number 3, not number 1. The correct, complete affiliation is: Unit of Molecular Mutagenesis and DNA Repair, National Institute for Cancer Research, IST, Genoa, Italy

