# Supplementary figures and images for: Noncanonical DNA Motifs as Transactivation Targets by Wild Type and Mutant p53
Source: PLoS Genet. 2008 Jun 27;4(6):e1000104. doi: 10.1371/journal.pgen.1000104 (PMC2518093; doi:10.1371/journal.pgen.1000104)

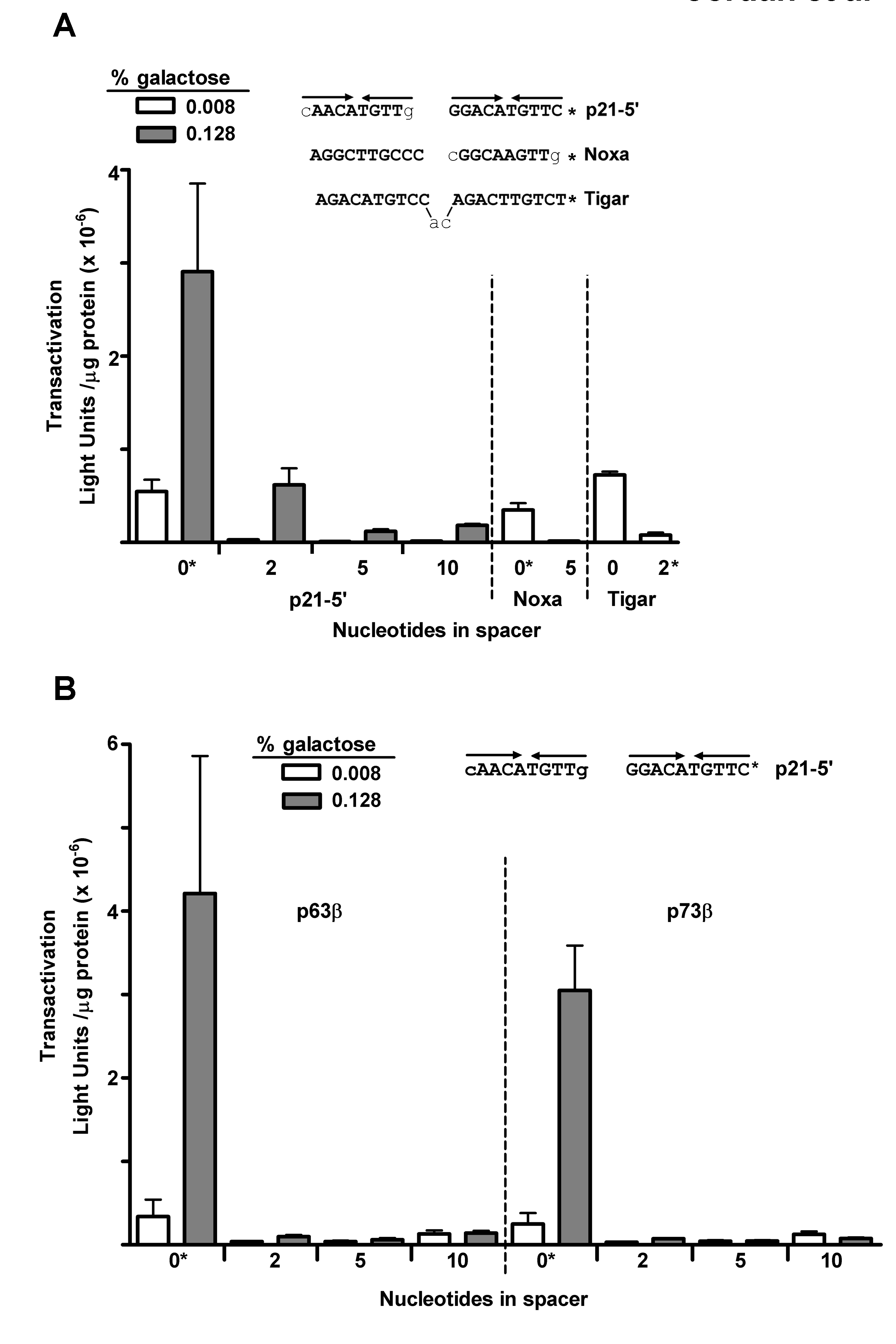

Supplement: Figure S2 — Increase in spacer decreases transactivation by p53 family members. (A) The plasmid-based haploid yeast system [30] was utilized to determine the extent to which p53 transactivation was impacted by a spacer between decamer half-sites. Transactivation was measured as relative light units/µg protein at 0.008% and 0.128% galactose and compared to WT p53 transactivation from the p21-5′ RE. Depicted are the average light units/µg protein and standard deviations from 3 biological repeats. The average light units/µg protein for WT p53 towards p21-5′ at 0.128% galactose was 2.9 million. (B) The transactivation capacity of p63β and p73β from the p21-5′ RE containing spacers of 0, 2, 5 and 10 nt was measured to determine the affect of spacers on transactivation by p53 family members. Transactivation was measured at moderate and high levels of p53 expression. (0.43 MB TIF) [file pgen.1000104.s002.tif]

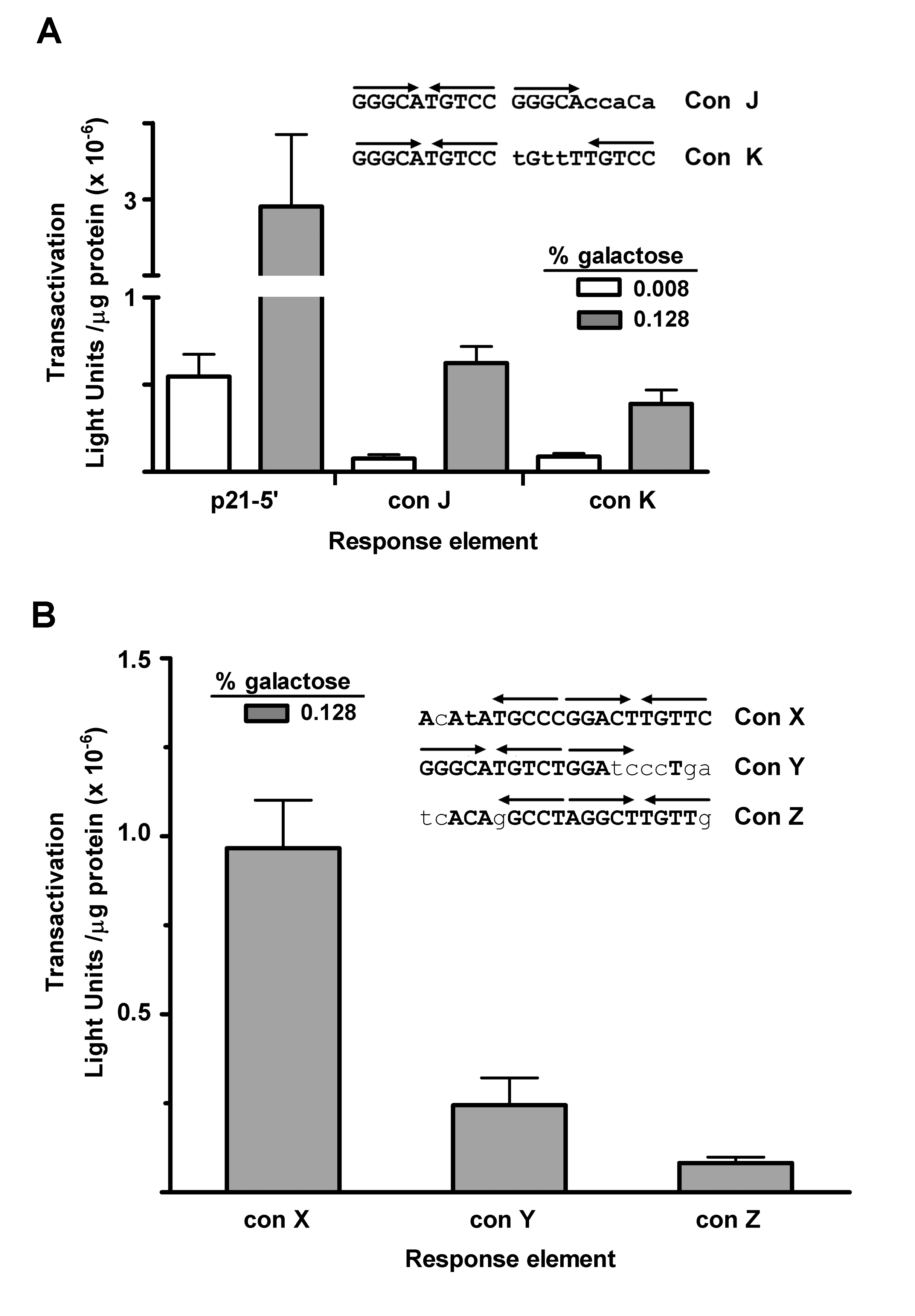

Supplement: Figure S3 — Transactivation from noncanonical 3/4-site REs in haploid yeast. (A) The ability of p53 to transactivate from the consensus 3/4-site REs, Con J and Con K were determined in the haploid yeast system, as described in Inga et al. [30]. The ability of WT p53 to transactivate from the 3/4-site REs was measured 24 hours post inoculation into the galactose-supplemented media by a luciferase assay. The ability of WT p53 to transactivate from each 3/4-site RE was compared to the ability of WT p53 to transactivate from the p21-5′ RE at 0.128% galactose. Transactivation is depicted as the light units/µg protein and presented as the mean and standard deviation of 3 independent experiments. The average light units/µg protein from p21-5′ at 0.128% galactose was 2.9 million. Solid arrows indicate a 1/4-binding site. (B) Sequence dependence of p53 transactivation from 3/4-site REs. The plasmid-based haploid yeast system [30] was utilized to determine the sequence requirements for transactivation from noncanonical 3/4-site REs at high expression levels (0.128% galactose). The central core “CWWG” motif was altered from CATG to CTTG and flanking regions were changed as described. Transactivation from 3/4-site REs was measured with a luciferase assay and calculated as light units/µg protein. (0.51 MB TIF) [file pgen.1000104.s003.tif]

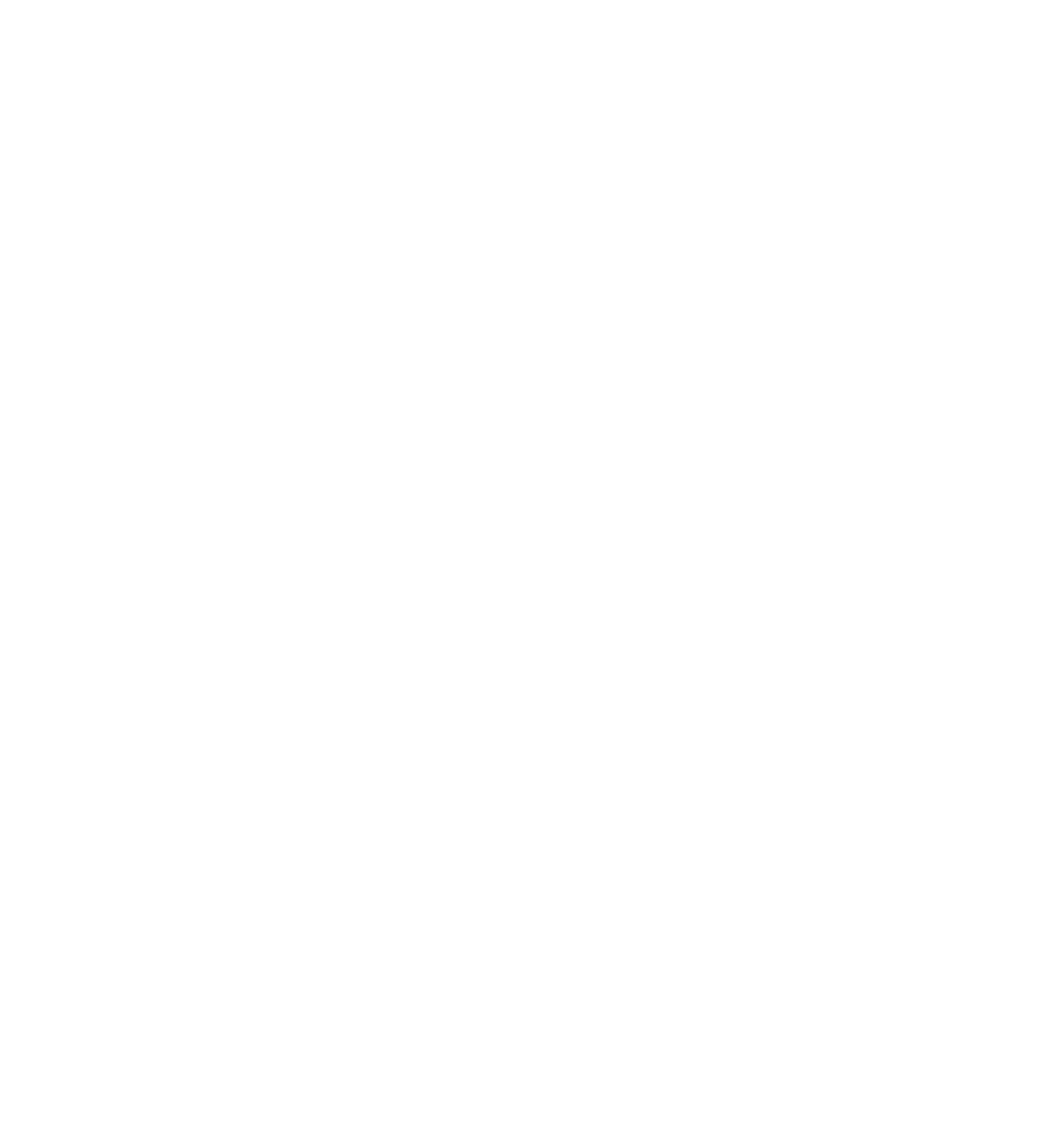

Supplement: Figure S4 — Mutant p53 expression. To exclude the possibility that variation in transactivation capacity towards the canonical and noncanonical REs by mutant p53 was due to variable protein levels, the relative expression of p53 mutants was compared to WT p53. Western analysis was performed 24 hours following transient transfection of mock, WT p53 or mutant p53 pCMV plasmid vectors into SaOS2 cells. Thirty-five µg of total protein was run on 4–12% BisTris NuPAGE as described in the Materials and Methods. Staining of the gel with SimplyBlue SafeStain (Invitrogen) for total loading protein was performed to discriminate possible overlap of protein between the loading control actin and the truncated p53 protein Q331stop which run at comparable distances. (2.98 MB TIF) [file pgen.1000104.s004.tif]
