# Supplementary material for: Noncanonical DNA Motifs as Transactivation Targets by Wild Type and Mutant p53
Source: PLoS Genet. 2008 Jun 27;4(6):e1000104. doi: 10.1371/journal.pgen.1000104 (PMC2518093; doi:10.1371/journal.pgen.1000104)
Supplement: Table S1 — Response element sequences. (0.05 MB DOC) [file pgen.1000104.s005.doc]

**Table S1. Response element sequences.**

| **Response Element** | **Sequence** | | |
| --- | --- | --- | --- |
|  | **Half-site** | **Spacer** | **Half-site** |
| **MDM2 RE1** | **GG**t**CAAGTT**g |  | **GGACA**c**GTCC** |
| **MDM2 RE2** | **GAGCTA**ag**TC** | c | t**GACATGTCT** |
| **MDM2 natural^a^** | **MDM2 RE1** | ggcgtcggctgtcggag | **MDM2 RE2** |
| **p21-5’^b^** | c**AACATGTT**g |  | **GGACATGTTC** |
| **Noxa^b^** | **AGGCTTGCCC** |  | c**GGCAAGTT**g |
| **TIGAR** | **AGACATGTCC** | ac | **AGACTTGTCT** |
| **p21-5’ left (L)** | c**AACATGTT**g |  |  |
| **P21-5’ right (R)** |  |  | **GGACATGTTC** |
| **con D** | **GGACATGCCC** |  |  |
| **con G** | **GGGCATGTCT** |  |  |
| **14-3-3σ site 1** | **AGGCATGT**g**C** |  | c**A**c**CATGCCC** |
| **con J** | **GGGCATGTCC** |  | **GGGCA**cca**C**a |
| **con K** | **GGGCATGTCC** |  | t**G**tt**TTGTCC** |
| **PIDD** | **AGGC**c**TGCCT** | gcgtgctg | **GGACATGTCT** |
| **PIDD no spacer** | **AGGC**c**TGCCT** |  | **GGACATGTCT** |
| **¾ PIDD-A** | ct**GC**g**TGCT**g |  | **GGACATGTCT** |
| **¾ PIDD-B** | **AGGC**c**TGCCT** |  | **G**c**G**tgctggg |
| **p21-3’** | **GGGCATGTCT** |  | **GGGCT**gaga**T** |
| **PCNA** | **GAACAAGTCC** |  | **GGGCAT**a**T**g**T** |
| **14-3-3σ site 2** | ta**G**c**A**t**TAGCCC** |  | **AGACATGTCC** |
| **APAF1** | **AGACATGTCT** | **GGA**g**A**ccctagga | **AGACATGTCC** |
| **con X** | **A**c**A**t**ATGCCC** |  | **GGACTTGTTC** |
| **con Y** | **GGGCATGTCT** |  | **GGAt**ccc**Tg**a |
| **con Z** | tc**ACA**g**GCCT** |  | **AGGCTTGTT**g |

^a^In the case of MDM2 natural, the natural site refers to two full sites that are separated by a 17 nucleotide spacer. Therefore, the half-site sequence refers to either of the full-site REs separated by the spacer (for sequences see MDM2 RE1 and MDM2 RE2).

^b^Sequences for spacers of increasing nucleotide length placed between half-sites: c, cc, ccc, ccct, ccctg.
